# Deep-blue emitting 9,10-bis(perfluorobenzyl)anthracene

**DOI:** 10.3762/bjoc.21.39

**Published:** 2025-03-07

**Authors:** Long K San, Sebastian Balser, Brian J Reeves, Tyler T Clikeman, Yu-Sheng Chen, Steven H Strauss, Olga V Boltalina

**Affiliations:** 1 Department of Chemistry, Colorado State University, Fort Collins, CO 80523, USA; 2 ChemMatCARS, University of Chicago Advanced Photon Source, Argonne, IL 60439, USA

**Keywords:** anthracene, dibromoanthracene, electron poor polyaromatic systems, fluorescence, perfluoroalkylation, perfluorobenzylation, photochemistry

## Abstract

A new deep-blue emitting and highly fluorescent anthracene (ANTH) derivative containing perfluorobenzyl (Bn_F_) groups, 9,10-ANTH(Bn_F_)_2_, was synthesized in a single step reaction of ANTH or ANTH(Br)_2_ with Bn_F_I, using either a high-temperature Cu-/Na_2_S_2_O_3_-promoted reaction or via a room-temperature photochemical reaction. Its structure was elucidated by NMR spectroscopy and single crystal X-ray diffractometry. The latter revealed no π–π interaction between neighboring ANTH cores. A combination of high photoluminescence quantum yield (PLQY) of 0.85 for 9,10-ANTH(Bn_F_)_2_, its significantly improved photostability compared to ANTH and 9,10-ANTH derivatives, and a simple synthetic access makes it an attractive material as a deep-blue OLED emitter and an efficient fluorescent probe.

## Introduction

The revolution of small organic molecules in the semiconductor industry continues to progress, replacing some silicon and metal-based electronic components. Acenes, such as anthracene (ANTH) and its higher homologues, represent some of the most studied materials that have promising applications in optoelectronics. For example, investigation of the photophysical and electronic properties of ANTH began in the 1960s [1–3], and since then many new ANTH derivatives have been synthesized and explored for their use as fluorescent probes, organic semiconductors, and emitters for organic light emitting diodes (OLEDs) [4–8]. More recently, an increased interest in the studies of triplet–triplet annihilation mechanisms in anthracene emitter materials [9] and the design of efficient OLED emitters based on hyperfluorescence [10–11], which could exhibit high-purity color and improved stability, has been observed.

OLED materials have attracted considerable research because they found commercial uses for flat panel displays and solid-state lighting applications. Currently, the design of efficient and highly stable blue fluorescent emitters remains a challenge for material scientists [12–14]. One of the structural features favorable for an efficient anthracene-based blue emitter is the introduction of bulky substituents in the 9 and 10 positions, which results in a solid-state packing with limited π–π intermolecular interactions, which, in turn, tends to suppress undesirable fluorescence self-quenching [15]. Not only do bulky substituents disrupt intermolecular interactions of this type, they can also provide higher chemical stability and reduce or prevent the photodimerization and photo-oxidation to which all acenes are prone [13]. Furthermore, bulky fluorinated substituents, such as perfluoroalkyls and perfluoroaryls, have been predicted by DFT [16] and shown experimentally [17], to improve the air- and photostability of acene-containing materials, by increasing their electron affinity and/or by sterically blocking reactive sites, respectively.

In this work, we explored the effects of the bulky and electron-withdrawing substituent perfluorobenzyl (Bn_F_) on the photophysical properties and on the air- and photostability of ANTH for the first time. We hypothesized that the steric bulk of Bn_F_ and the flexibility of the –CF_2_– moiety between ANTH and the –C_6_F_5_ group might provide the desired spatial isolation of the ANTH (and other PAH) cores, and result in enhanced photoluminescence by disrupting close π–π stacking in the solid state. In addition, air- and photostability might also be improved due to the steric and electronic properties of multiple Bn_F_ substituents. For example, *C*_5_-pentakis(Bn_F_)corannulene (*C*_5_-CORA(Bn_F_)_5_) [18–19] exhibited improved acceptor strength relative to unsubstituted CORA due to the electron-withdrawing Bn_F_ groups (which are similar to the electron-withdrawing properties of CF_3_ groups [18–19]). In addition, the size and conformational flexibility of the five Bn_F_ groups resulted in significant disruption of CORA–CORA π–π interactions due to the greater CORA–CORA bowl-to-bowl distances relative to the less bulky *C*_5_-CORA(CF_3_)_5_ analogue [18–19]. However, corannulene derivatives are only weakly fluorescent [20–21] and are not suitable as emitters for OLEDs or fluorescent probes. A family of perfluorobenzylated derivatives of another PAH, viz. perylene (PERY), was recently reported by our group [22]. We showed that the acceptor strength of PERY(Bn_F_)*_n_* derivatives (*n* = 1–5) significantly increased with increasing *n* [22].

In this work, we report the first synthesis of ANTH(Bn_F_)*_n_* derivatives (*n* = 1, 2), molecular and solid-state structures, and their photophysical properties.

## Results and Discussion

### Synthesis

Perfluoroalkylation of anthracene has been previously achieved by bottom-up syntheses [23], three-step reactions with anthraquinone starting materials [24], or by Ullman coupling reactions of acene bromides with perfluoroalkyl iodides [25]. Notably, direct UV photoinduced reactions between anthracene and perfluoroalkyl iodides in the presence of a reducing agent, Na_2_S_2_O_3_, yielded perfluoroalkylated dimeric molecules, instead of expected 9,10-(perfluoroalkyl)_2_ANTH [26].

To the best of our knowledge, perfluorobenzylation of anthracene has not been reported prior to this study. The only PAH perfluorobenzylations were reported by our group: with CORA using gas-phase solvent-free reactions in sealed ampules and with PERY using solution-phase reactions at elevated temperatures in the presence of Cu metal or another reducing agent [22].

**Scheme 1:**
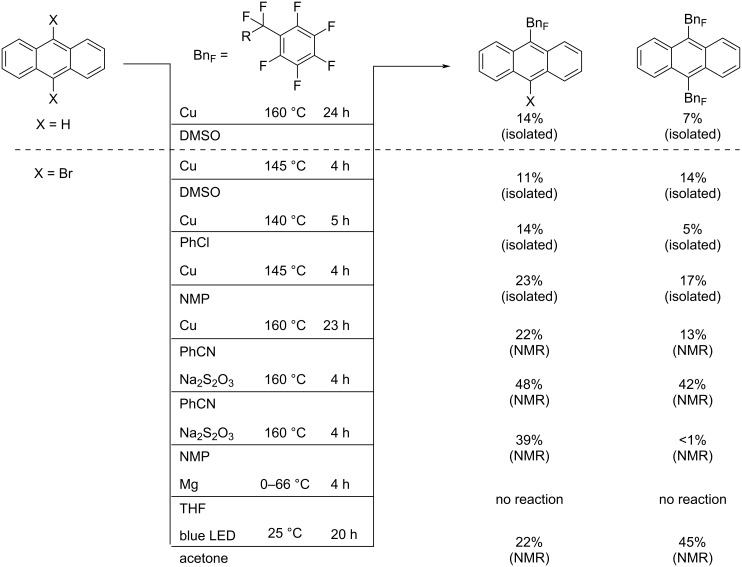
List of reactions, experimental conditions and yields studied in this work.

In this study we first explored the approaches from our previous work: a high-temperature gas-phase reaction between ANTH and Bn_F_I [18–19], and a solution-phase reaction in the presence of Cu in DMSO [22]. The former approach yielded a complex, inseparable mixture of products and was not studied further. The latter approach afforded two isolable main products, 9-ANTH(Bn_F_) (14% yield) and 9,10-ANTH(Bn_F_)_2_ (7% yield, both are isolated yields based on ANTH). Several other, yet to be identified, ANTH(Bn_F_)*_n_* compounds were also observed. In this reaction, ANTH dissolved in DMSO was heated to between 120 °C and 160 °C in the presence of 2 equiv of Bn_F_I and 3 equiv of Cu powder as promotor for 24 h, resulting in the complete conversion of ANTH to reaction products. When 10 equiv Bn_F_I was used instead, a 14% isolated yield of 9,10-ANTH(Bn_F_)_2_ (based on ANTH) was achieved (Scheme 1).

Due to these rather low yields, a change in the substrate from ANTH to 9,10-ANTH(Br)_2_ was investigated. According to the literature [25], the use of halogenated substrates can result in higher selectivity with milder reaction temperatures. The weaker C(sp^2^)–Br bond is easier to cleave than a C(sp^2^)–H bond, resulting in a higher likelihood of radical substitution at those positions. To the best of our knowledge, there are no previous examples of perfluorobenzyl substitution of any PAH(Br)*_n_* starting material.

A series of reactions with 9,10-ANTH(Br)_2_ and Bn_F_I in the presence of Cu were carried out in four high-boiling organic solvents: DMSO, chlorobenzene (PhCl), benzonitrile (PhCN), and *N*-methylpyrrolidinone (NMP). When PhCl was the solvent, the 14% yield of the mono-substituted product was comparable to that in the reaction of unsubstituted ANTH and Bn_F_I in DMSO. When NMP was the solvent, a slight increase in the yield of of 9,10-ANTH(Bn_F_)_2_, 17%, was achieved relative to the reactions in DMSO and PhCl.

To further improve the yield of 9,10-ANTH(Bn_F_)_2_, the experimental conditions used for the perfluorobenzylation of PERY were used [22]. 9,10-ANTH(Br)_2_ and Bn_F_I were dissolved and heated to 160 °C in PhCN in the presence of either Cu powder (22 h) or Na_2_S_2_O_3_ (4 h). According to the ^1^H NMR spectra of the filtered product mixtures, the reaction with Cu showed poor conversion, but the reaction with Na_2_S_2_O_3_ resulted in a 2:1 mixture of 9-Br-10-Bn_F_-ANTH and 9,10-ANTH(Bn_F_)_2_ with no remaining 9,10-ANTH(Br)_2_. However, the high boiling point of PhCN and its miscibility with almost every solvent that could be used for liquid–liquid extraction made the determination of isolated yields of the solid products practically impossible. When NMP was used as the solvent, the reaction of 9,10-ANTH(Br)_2_, Bn_F_I, and Na_2_S_2_O_3_ did not result in the formation of 9,10-ANTH(Bn_F_)_2_ but mainly in the formation of 9-Br-10-Bn_F_-ANTH (39%, determined by NMR).

Overall, the methods described above all suffered from the same disadvantages: low yields and challenging workups of the product mixtures due to the high boiling points of the solvents used: NMP (202 °C), PhCN (190 °C), DMSO (189 °C), and PhCl (132 °C). An attempt was made to synthesize ANTH(Bn_F_)_2_ starting with ANTH(Br)_2_ via a Grignard approach with Bn_F_I. While it was possible to prepare ANTH(MgBr)_2_, the desired product was not formed.

Therefore, a photochemical method for the synthesis of 9,10-ANTH(Bn_F_)_2_ was investigated. Photochemical perfluoroalkylation reactions have attracted attention since 2011 because milder reaction conditions can be used to achieve higher yields [27]. Even in the absence of a transition-metal-based photosensitizer, a recent study showed that perfluoroalkylation using perfluoroalkyl iodides (R_F_I) could be carried out by activation of the R_F_–I bonds by formation of electron donor–electron acceptor complexes with an organic base such as tetramethylethylenediamine (TMEDA) or 1,8-diazabicyclo(5.4.0)undec-7-ene (DBU) [28].

Our initial attempt to apply this method was as follows: A reaction mixture of 9,10-ANTH(Br)_2_, 10 equiv Bn_F_I, and 10 equiv DBU dissolved in acetone was illuminated with a blue LED for 20 h (see Figures S1 and S2 in Supporting Information File 1). The ^1^H NMR analysis of the crude reaction mixture indicated 60% conversion of 9,10-ANTH(Br)_2_ and a 1:1 ratio of only two products, 9,10-ANTH(Bn_F_)_2_ and 9-Br-10-Bn_F_-ANTH. Even after only 2 h, it was possible to observe both products by ^1^H NMR, indicating a relatively fast and efficient conversion into the desired products. Further optimization of this promising photochemical method is currently under way in our laboratory.

### Isolation and characterization

**Figure 1:**
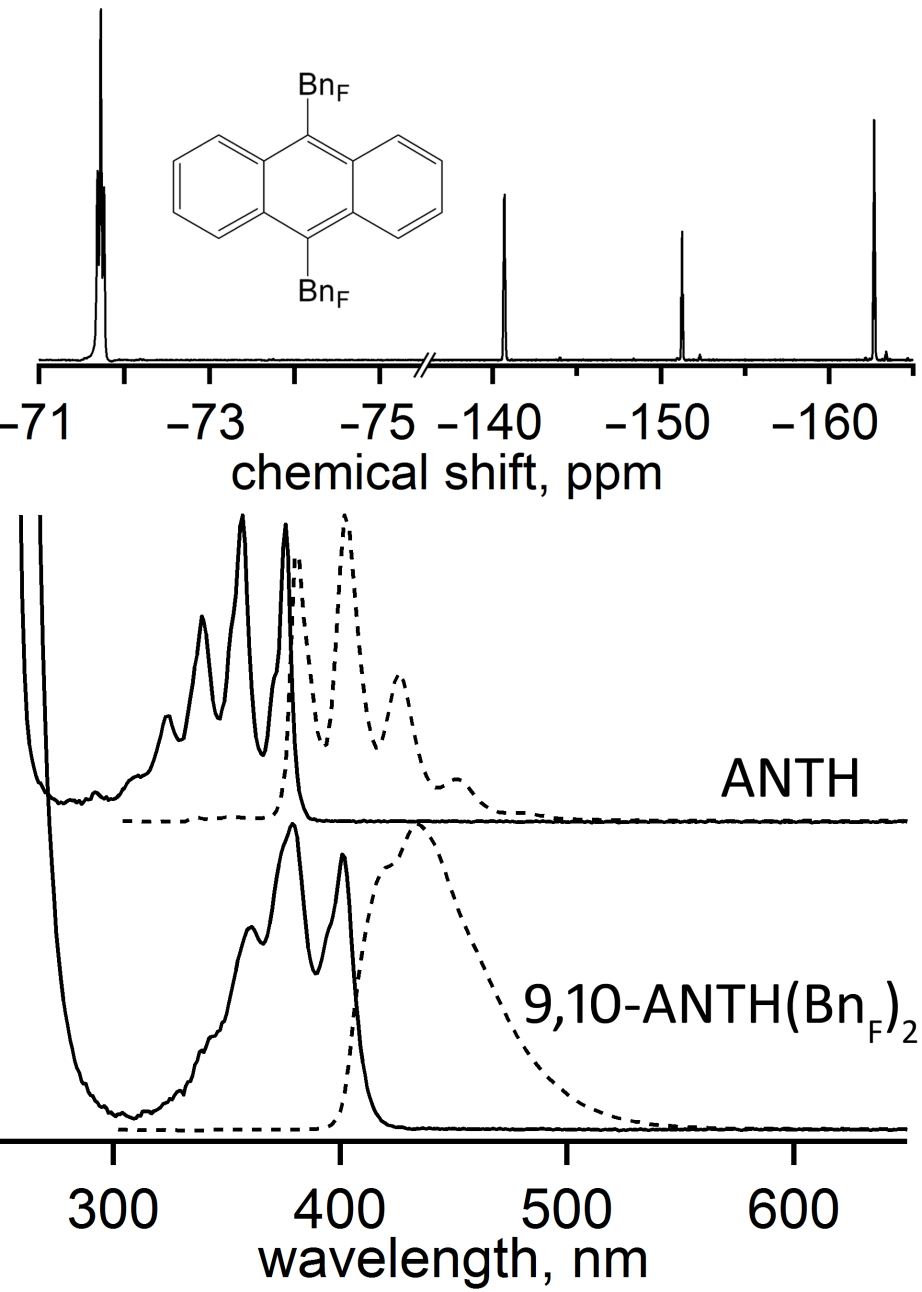
Top: 379 MHz ^19^F NMR spectrum of 9,10-ANTH(Bn_F_)_2_ in CDCl_3_. Bottom: absorption (aerobic, solid line) and emission (anaerobic, dashed line) spectra in cyclohexane.

Pure samples of 9,10-ANTH(Bn_F_)_2_ and 9-ANTH(Bn_F_) were isolated by HPLC (Figure S3, Supporting Information File 1). In the ^19^F NMR spectra of 9,10-ANTH(Bn_F_)_2_ (Figure 1) and 9-ANTH(Bn_F_) (Figure S4 in Supporting Information File 1), only four resonances in a 2:2:1:2 ratio were observed, commensurate with the previously reported data for compounds bearing Bn_F_ groups [18,29]. The ^19^F resonances of the CF_2_ and C_6_F_5_ moieties appeared in the expected regions of −δ = 71–74 ppm and −δ = 140–165 ppm, respectively. Through-space F–F coupling was observed between the CF_2_ group and the *ortho*-F atoms, resulting in a triplet and a multiplet, respectively. Using 1,4-C_6_H_4_(CF_3_)_2_ as an internal standard, the F/H mole ratio was found to be 1.74 (theoretical = 1.75), confirming the composition to be ANTH(Bn_F_)_2_.

**Figure 2:**
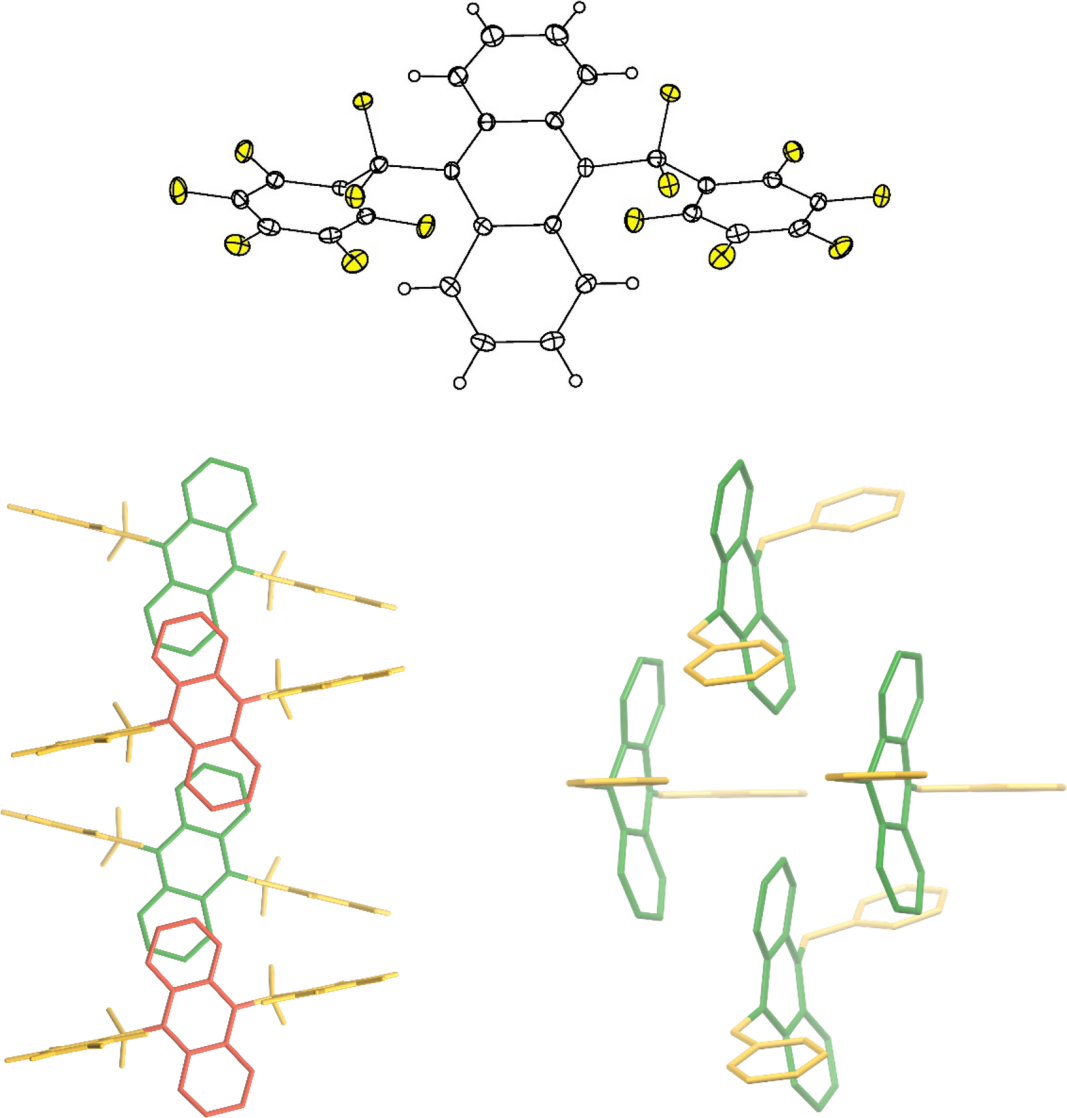
Top: X-ray structure of 9,10-ANTH(Bn_F_)_2_, thermal ellipsoids 50% probability. Bottom: a view down the crystallographic *c*-axis (left) and an off-side view (right) of the solid-state packing in the X-ray structure of 9,10-ANTH(Bn_F_)_2_. Two different stacked columns of ANTH moieties are colored red and green, Bn_F_ groups are colored in yellow, and H atoms have been omitted for clarity. In the off-side view (bottom right), both, F and H atoms are omitted for a better overview.

Slow evaporation of a CH_2_Cl_2_ solution of 9,10-ANTH(Bn_F_)_2_ at 2 °C afforded off-white plates suitable for X-ray diffraction. The structure shown in Figure 2 (top panel) confirmed the 9,10-substitution pattern. Interestingly, only one conformer is observed, with both perfluorobenzyl groups pointing in the same direction (instead of the opposite directions) on the ANTH core, in contrast with the earlier reported by us presence of two different conformers of perfuorobenzylated corannulene in the single crystal, which provides yet another example of the structural flexibility of perfluorobenzylated compounds and their sensitivity towards chemical environment [18]. The molecules are arranged in two distinct columns, colored red and green that are skewed from one another by 31.2°.

There is no significant π–π overlap between the ANTH cores. The ANTH hexagons that appear to be overlapped (see Figure 2, bottom left) are separated by 4.154 Å and are tilted from each other by 21.6°. The columns of ANTH cores are insulated along the crystallographic *b*-axis by the Bn_F_ groups, which can be more clearly seen by viewing down the long ANTH axis (Figure S5 in Supporting Information File 1). This insulation further inhibits any electronic coupling between the columns. Having effectively zero π–π overlap should result in reduced electronic coupling between molecules, which may be a beneficial factor for OLED applications because isolated electronically excited molecules are more likely to undergo fluorescence instead of non-radiative relaxation, as discussed in some literature [13]. At the same time, it should be noted that emission properties in the crystalline phase and amorphous films, which are often used in OLED devices, can differ and this requires further studies.

### Photophysical properties

Absorption and emission spectra of ANTH and 9,10-ANTH(Bn_F_)_2_ are shown in Figure 1. The maximum λ_abs_ and λ_em_ values for ANTH and the 9,10-disubstituted ANTH derivatives are shown in Table 1. The maximum λ_em_ of 9,10-ANTH(Bn_F_)_2_ at 416 nm is the most red-shifted from ANTH of the listed compounds, resulting in deeper blue fluorescence. It also has the largest Stokes shift of 837 cm^−1^ (large Stokes shifts are attributed to attenuation of fluorescence by self-quenching processes [30]). Only ANTH obeys the mirror image rule, whereas 9,10-ANTH(Bn_F_)_2_ loses vibronic structure in the emission spectrum. This may be the result of different conformations upon relaxation [31].

The HOMO–LUMO gap (*E*_g_) decreases from ANTH (3.28 eV) to 9,10-ANTH(Bn_F_)_2_ (3.05 eV). A decrease in *E*_g_ is regarded as one of the methods for improving the efficiencies in OLED performance, since lower applied voltages will be required. It has been shown that a desired applied voltage should be below 10 V to obtain significant light intensity from ANTH-based OLEDs [8]. Such low operating voltage may reduce power consumption and increase the lifetime of a blue-emitting device.

The photophysical data of ANTH and 9,10-disubstituted anthracene derivatives are summarized in Table 1. The measured photoluminescence quantum yield, PLQY (Φ_f_) of ANTH in cyclohexane is in agreement with the literature values of 0.28–0.36 [32–35]. An increase in Φ_f_ was previously observed for 9,10-ANTH(X)_2_ derivatives bearing fluorine-containing electron-withdrawing groups. For example, the Φ_f_ values increased from 0.28 for ANTH to 0.54 (X = F) to 0.68 (X = CF_3_) which is similar to that of X = C_6_F_5_ [32]. To our knowledge, 9,10-ANTH(Bn_F_)_2_ has the second highest Φ_f_ value reported for ANTH derivatives with fluorinated substituents (the highest is Φ_f_ = 0.97 for 9,10-ANTH(C_8_F_17_)_2_) [25].

**Table 1:** Relative PLQY (Φ_f_), absorption and emission maxima, and Stokes’ shifts in cyclohexane.

compound	Φ_f_	λ_abs_ (nm)	λ_em_ (nm)	Δλ (cm^−1^)	ref

ANTH	0.40^a^	376	381	349	this work
9,10-ANTH(Bn_F_)_2_	0.85^a^	402	416	837	this work
9,10-ANTH(F)_2_^b^	0.54^a^	393	394	65	[32]
9,10-ANTH(CF_3_)_2_^b^	0.68^a^	400	410	610	[32]
9,10-ANTH(C_6_F_5_)_2_^b^	0.66^a^	390	400	641	[32]
9,10-ANTH(C_8_F_17_)_2_^c,d^	0.97	409	413	237	[25]

^a^Standard: quinine sulfate (Φ_f_ = 0.55); ^b^Φ_f_ (ANTH) = 0.28; ^c^Φ_f_ (ANTH) = 0.27 (EtOH, ref [3])); ^d^standard: 9,10-ANTH(Ph)_2_ (Φ_f_ = 0.90).

Several comparative photostability studies for unsubstituted ANTH and ANTH derivatives using UV–vis spectroscopy have been reported, in which the authors studied, if photostability enhancements were achieved due to chemical modification of ANTH [25,32]. Changes in the UV–vis spectra were typically used as an indication of the compounds’ decomposition. It is well established that ANTH is rapidly oxidized to form anthraquinone upon exposure to light in the presence of oxygen. Furthermore, endoperoxides were shown to form from ANTH(R_F_)*_n_* derivatives within minutes at room temperature in the presence of oxygen when irradiated with a high pressure mercury lamp [32]. When 9,10-bis(perfluorooctyl)anthracene was dissolved in CHCl_3_ and irradiated for 350 minutes, photodecomposition was observed. However, the use of fluorous solvents was shown to dramatically improve its photostability [25].

**Figure 3:**
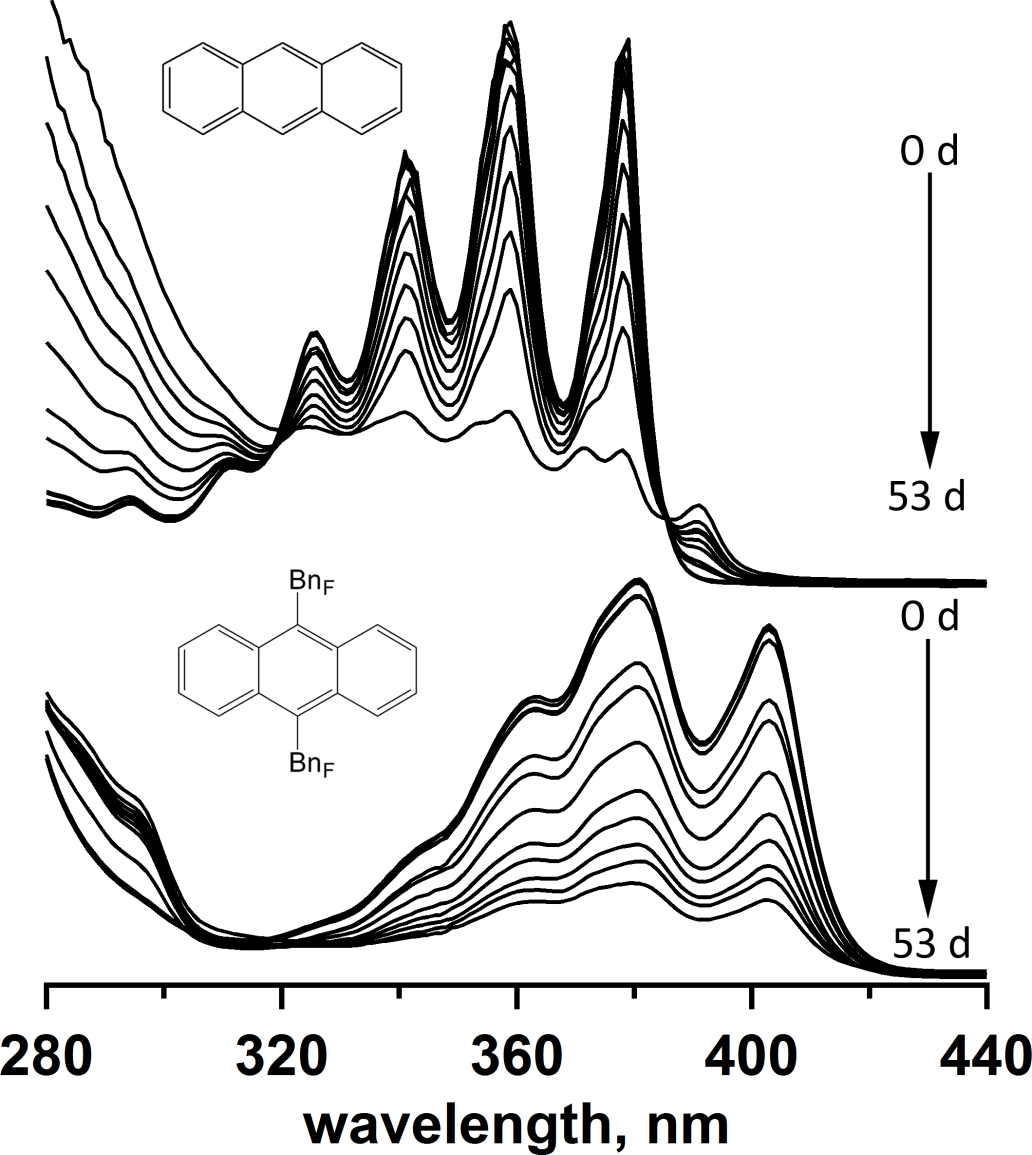
Absorption spectra of ANTH and 9,10-ANTH(Bn_F_)_2_ in CH_2_Cl_2_ recorded over the period of 53 days in air and under irradiation with incandescent light.

We performed a comparative study of the photostability of ANTH and 9,10-ANTH(Bn_F_)_2_ dissolved in CH_2_Cl_2_. The absorbance was measured over 53 days in the presence of oxygen, as shown in Figure 3. Both samples were irradiated with a 34 W incandescent bulb. The UV–vis spectra of ANTH monitored over time showed that new absorbance features began to appear, suggesting the formation of new products. In contrast, the UV–vis spectra of 9,10-ANTH(Bn_F_)_2_ showed no new absorbance features over time, suggesting that either photoproducts were insoluble resulting in decreased concentration of 9,10-ANTH(Bn_F_)_2_ in the irradiated solution or new photoproducts had similar absorption features.

To identify the photoproducts in the irradiated solutions of ANTH and 9,10-ANTH(Bn_F_)_2_, complementary NMR analyses were carried out (Figures S6 and S7 in Supporting Information File 1). The ^1^H NMR spectrum of the photoirradiated ANTH solution showed that ANTH was no longer present and that small amounts of anthraquinone and other unidentified products (presumably other oxidized species) had formed. In contrast, the ^1^H NMR spectrum of the photoirradiated 9,10-ANTH(Bn_F_)_2_ solution contained only resonances for the starting material (20%) and for a single presumed photoproduct (80%) which retained the symmetry of 9,10-ANTH(Bn_F_)_2_ (see Supporting Information File 1, Figures S7 and S9 (^19^F NMR)). An HPLC analysis of the photoirradiated ANTH solution revealed that at least three compounds were present, in accordance with an earlier report [32]. In the photoirradiated 9,10-ANTH(Bn_F_)_2_ sample, the HPLC chromatogram shown in Figure S10 (Supporting Information File 1) showed 9,10-ANTH(Bn_F_)_2_ (*t*_R_ = 4.5 minutes) and a major product (*t*_R_ = 6 minutes), in agreement with the NMR results.

**Figure 4:**
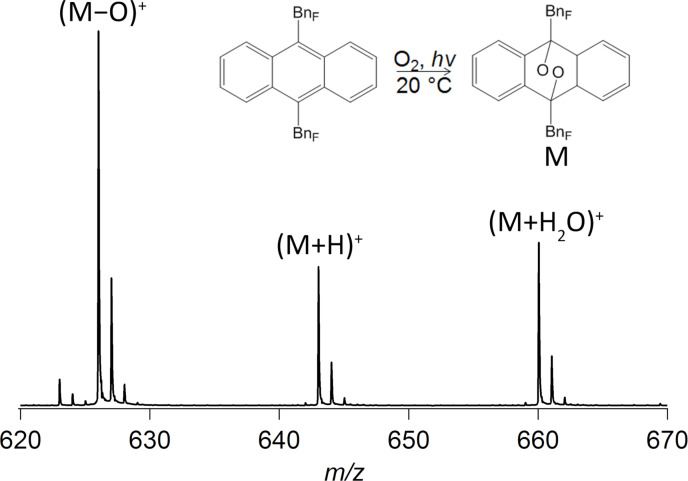
Direct analysis in real time (DART) positive ion mass spectrum of the photoirradiated 9,10-ANTH(Bn_F_)_2_ sample. M denotes molecular ion; the insert shows the reaction scheme for the formation of the endoperoxide.

The positive ion mass spectrum of the photoirradiated 9,10-ANTH(BnF)_2_ provides further insight into the composition of the main photoproduct. The presence of the protonated endoperoxide of 9,10-ANTH(Bn_F_)_2_ as the heaviest species in the mass spectrum, along with two fragments, allows a tentative conclusion that photooxidation proceeds via dearomatization of the central ring due to the formation of the endoperoxide (Figure 4). This suggestion is also supported by a recent work of Sun and co-workers where they observed and structurally characterized the endoperoxide of 9,10-ANTH(C_8_F_17_)_2_ [36].

These results make it clear that monitoring photoirradiated solutions by UV–vis spectroscopy must be accompanied by complementary analyses to enable accurate interpretation of the changes in the UV–vis spectra and identification of the photoproducts.

**Figure 5:**
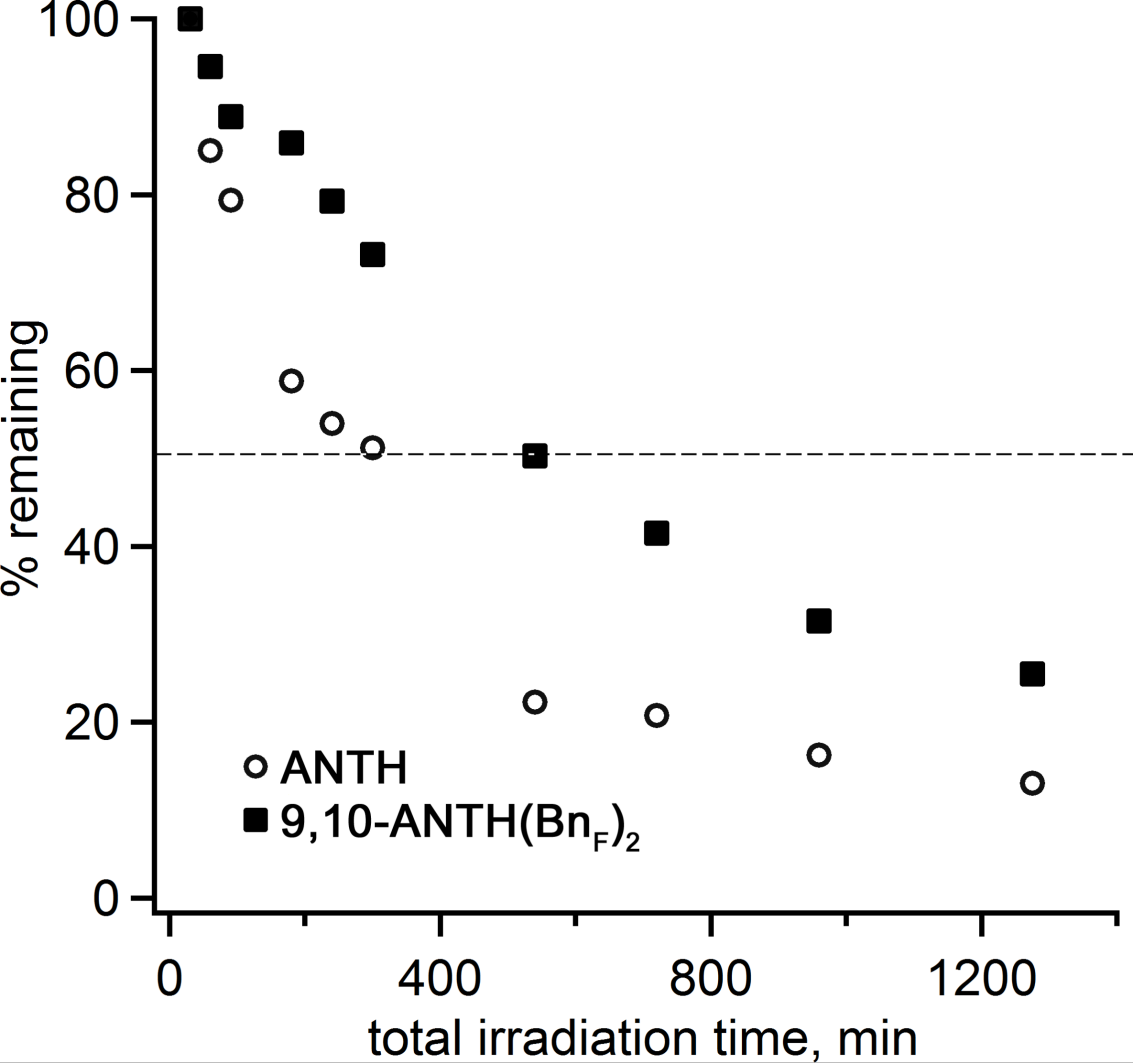
The % remaining of ANTH and 9,10-ANTH(Bn_F_)_2_ dissolved in CDCl_3_ upon irradiation. Resonances δ = 7.48 ppm for ANTH and δ = 7.46 ppm for 9,10-ANTH(Bn_F_)_2_ were normalized against the resonance for residual CHCl_3_. The dashed line indicates 50% remaining.

The next comparative photostability experiment was carried out under anaerobic conditions. In that experiment, a sample of 9,10-ANTH(Bn_F_)_2_ was dissolved in CDCl_3_ in an NMR tube, degassed with three freeze-pump-thaw cycles, and irradiated with a high-pressure mercury arc lamp. The ^1^H NMR integrated intensity of the resonance at δ = 7.46 ppm, normalized against the resonance for residual CHCl_3_, was monitored as a function of irradiation time. The results are shown in Supporting Information File 1, Table S1. These same sets of experimental conditions were applied to a sample of ANTH. A gradual decrease in the % remaining of both ANTH and 9,10-ANTH(Bn_F_)_2_ can be seen in Figure 5. After 240 minutes of irradiation, 54% and 79% of ANTH and 9,10-ANTH(Bn_F_)_2_, respectively, were still present. After 540 minutes of irradiation, ANTH decreased to 22% while 9,10-ANTH(Bn_F_)_2_ remained just above 50%. Clearly, the photostability of 9,10-ANTH(Bn_F_)_2_ is far greater than the parent ANTH molecule. Significantly, no new ^1^H NMR resonances appeared upon irradiation of the 9,10-ANTH(Bn_F_)_2_ solution, except for proton signals due to 9,10-ANTH(Bn_F_)_2_, indicating that no new soluble photoproducts are formed in the absence of oxygen.

On the other hand, during photoirradiation of the ANTH solution, a set of new resonances (δ = 6.92 (m, 2H), 6.81 (m, 2H), and 4.55 (s, 1H) ppm) appeared and increased in intensity relative to the ANTH resonances. The new photoproduct was identified as a dimer, dianthracene, which had previously been shown to form under anaerobic UV irradiation [37]. Notably, the apparent absence of the photodimer in the case of 9,10-ANTH(Bn_F_)_2_ is likely due to the steric hindrance of the bulky perfluorobenzyl groups in the 9- and 10-positions on the ANTH core.

## Conclusion

The perfluorobenzylation of ANTH and ANTH(Br)_2_ was performed for the first time. The new compounds 9-ANTH(Bn_F_) and 9,10-ANTH(Bn_F_)_2_ were prepared in varying amounts using several synthetic procedures, including conventional high-temperature Cu-/Na_2_S_2_O_3_-promoted reactions and a room-temperature photochemical reaction. The former approach resulted in challenging work-ups, low yields, and poor selectivity. The latter approach was shown to be more promising due to a faster rate, use of an inexpensive, low-boiling solvent, and easier work-up. This method will continue to be investigated in our laboratory. The emission spectrum of 9,10-ANTH(Bn_F_)_2_ revealed its deeper blue fluorescence relative to other 9,10-ANTH derivatives. These results, in conjunction with its high PLQY value (Φ_f_ = 0.85) and increased photostability, indicate that 9,10-ANTH(Bn_F_)_2_ may be useful for OLED applications and beyond, either as is or as a unique building block for development of more advanced derivatives.

## Experimental

### Solvents and reagents

The following reagents and solvents were used as received unless otherwise indicated: anthracene (TCI America, 94%); 9,10-dibromoanthracene (Thermo Scientific Chemicals, 98%); heptafluorobenzyl iodide (C_6_F_5_CF_2_I, SynQuest, 90%); cyclohexane (Mallinckrodt); 1,4‐bis(trifluoromethyl)benzene (1,4-C_6_H_4_(CF_3_)_2_, Central Glass Co., 99%); dimethyl sulfoxide (DMSO, Fisher Scientific, ACS grade); anhydrous magnesium sulfate (MgSO_4_, Fisher Scientific); Cu powder (Strem Chemicals, 99%); dichloromethane (EMD Chemicals, ACS grade); acetone (technical grade); THF (Aldrich/Merck, ACS grade, dried over 4 Å molecular sieves); quinine hemisulfate salt monohydrate (Fluka); sulfuric acid (EMD Chemicals); diethyl ether anhydrous (EMD Chemicals, ACS grade); chloroform-*d* (CDCl_3_, Cambridge Isotope Labs, 99.8%); hexafluorobenzene (C_6_F_6_, Oakwood Products); deionized distilled water (purified with a Barnstead NANOpure Ultrapure Water system, producing water with a final resistance of at least 18 MΩ); and silica gel (Sigma-Aldrich, 70–230 mesh, 60 Å). For HPLC separations: acetonitrile (Fisher Scientific ACS grade); toluene (Fisher Scientific, ACS grade); and heptane (Mallinckrodt Chemicals, ACS grade) were used as received.

### Instrumentation

^19^F (376 MHz) and ^1^H (400 MHz) NMR spectra were recorded using a Varian INOVA 400 instrument with a trace amount of C_8_H_4_F_6_ (δ(^19^F) = −66.35 ppm) added as the internal standard. UV–vis spectra were recorded using a Cary 500 UV–VIS–NIR. Emission spectra of the LED strips used in this study were measured with a Spectryx SpectryxBlue spectrometer. Fluorescence spectra were recorded using an AVIV ATF‐105 Auto‐Titrating Differential/Ratio Spectrofluorimeter with 90° measurement geometry.

### Synthesis

#### Method 1 (thermal, solution-phase reactions)

**Method 1.1 (ANTH, Cu, DMSO):** CF_2_C_6_F_5_I (19 µL, 0.11 mmol) was syringed into a glass ampoule containing anthracene (10 mg, 0.056 mmol) and copper powder (11 mg, 0.17 mmol) dissolved in DMSO, and degassed by a freeze-pump-thaw technique (3 × 10 min). The ampoule was then heated in an oil bath at 160 °C for 24 h. The reaction contents were extracted with Et_2_O and washed four to six times with doubly distilled water removing the aqueous layer each time. Anhydrous MgSO_4_ was added to the organic layer and passed through silica gel with DCM as the eluent. The organic layer was then concentrated to dryness. Yield (isolated): 9-H-10-Bn_F_-ANTH (14%), 9,10-(Bn_F_)_2_-ANTH (7%).

**Method 1.2 (9,10-ANTH(Br)_2_, Cu, DMSO):** 9,10-ANTH(Br)_2_ (13.4 mg, 39.8 µmol) was mixed with Cu powder (100 mg, 1.57 mmol) in a 10 mL rotavis reaction tube fitted with a wired septa in a glove box. DMSO (5 mL) was added to the reaction tube and the mixture was degassed with N_2_. The mixture was subsequentially heated to 145 °C. Once reflux was achieved, CF_2_C_6_F_5_I (34 µL, 207 µmol) was added dropwise via syringe. After 4 h, the reaction was removed from the oil bath, covered in Al foil and allowed to cool to room temperature. The reaction mixture was filtered to remove the Cu powder. The solvents were removed in vacuo producing an orange oil. 9,10-ANTH(Bn_F_)_2_ was isolated via multiple stages of HPLC separation. Yield (isolated): 9-Br-10-Bn_F_-ANTH (11%), 9,10-(Bn_F_)_2_-ANTH (14%).

**Method 1.3 (9,10-ANTH(Br)_2_, Cu, PhCl):** 9,10-ANTH(Br)_2_ (13.4 mg, 39.8 µmol) was mixed with Cu powder (100 mg, 1.57 mmol) in a 10 mL round-bottomed flask in a glove box. PhCl (5 mL) was added to the round-bottomed flask and a reflux condenser was affixed. The mixture was freeze-pump-thawed (3 × 10 min) and subsequentially heated to reflux. Once reflux was achieved, CF_2_C_6_F_5_I (68 µL, 414 µmol) was added via syringe. After 5 h, the reaction was removed from the oil bath, covered in Al foil and allowed to cool to room temperature. The reaction mixture was filtered to remove the Cu powder. The solvents were removed in vacuo producing an orange oil. 9,10-ANTH(Bn_F_)_2_ was isolated via two stages of HPLC separation. Yield (isolated): 9-Br-10-Bn_F_-ANTH (14%), 9,10-(Bn_F_)_2_-ANTH (5%).

**Method 1.4 (9,10-ANTH(Br)_2_, Cu, NMP):** 9,10-ANTH(Br)_2_ (13.4 mg, 39.8 µmol) was mixed with Cu powder (100 mg, 1.57 mmol) in a 10 mL rotavis reaction tube fitted with a wired septum in a glove box. NMP (5 mL) was added to the reaction tube and the mixture was degassed with N_2_. The mixture was subsequentially heated to 145 °C. Once reflux was achieved, CF_2_C_6_F_5_I (34 µL, 207 µmol) was added dropwise via syringe. After 4 h, the reaction was removed from the oil bath, covered in Al foil and allowed to cool to room temperature. The reaction mixture was filtered to remove the Cu powder. The solvents were removed in vacuo producing an orange oil. 9,10-ANTH(Bn_F_)_2_ was isolated via one stage of HPLC separation. Yield (isolated): 9-Br-10-Bn_F_-ANTH (23%), 9,10-(Bn_F_)_2_-ANTH (17%).

**Method 1.5 (9,10-ANTH(Br)_2_, Cu, PhCN):** Cu (50 mg, 0.79 mmol) was added to a 50 mL Schlenk flask containing PhCN (20 mL). 9,10-ANTH(Br)_2_ (42 mg, 125 µmol) and CF_2_C_6_F_5_I (97.5 µL, 625 µmol) were added. The mixture was freeze-pump-thawed (3 × 10 min) before being subsequentially heated to 160 °C for 23 h. After letting the reaction mixture cool to room temperature, the reaction contents were extracted with DCM and washed with H_2_O (6 × 10 mL), brine (1 × 10 mL), and dried over MgSO_4_. The solvents were removed in vacuo. It was attempted to remove leftover PhCN via azeotropic distillation with iPrOH and 1-butanol, but a total removal was not possible. Yield (NMR): 9-Br-10-Bn_F_-ANTH (22%), 9,10-(Bn_F_)_2_-ANTH (13%).

**Method 1.6 (9,10-ANTH(Br)_2_, Na_2_S_2_O_3_, PhCN):** In a 100 mL Schlenk flask, 9,10-ANTH(Br)_2_ (42 mg, 125 µmol) and Na_2_S_2_O_3_ (1.24 g, 5.00 mmol) were dissolved in PhCN (10 mL). In a separate Schlenk flask, CF_2_C_6_F_5_I (200 µL, 1.22 mmol) was dissolved in PhCN (30 mL). Both solutions were degassed via a freeze-pump-thaw technique (3 × 10 min). The 100 mL Schlenk flask was heated to 160 °C after which the CF_2_C_6_F_5_I solution was added dropwise through a drip funnel. Afterwards, the reaction mixture was kept at 160 °C for 4 h. After letting the reaction mixture cool to room temperature, the reaction contents were extracted with DCM and washed with H_2_O (6 × 10 mL), brine (1 × 10 mL), and dried over MgSO_4_. The solvents were removed in vacuo. It was attempted to remove leftover PhCN via azeotropic distillation with iPrOH and 1-butanol but a total removal was not possible. Yield (NMR): 9-Br-10-Bn_F_-ANTH (48%), 9,10-(Bn_F_)_2_-ANTH (42%).

**Method 1.7 (9,10-ANTH(Br)_2_, Na_2_S_2_O_3_, NMP):** In a 100 mL Schlenk flask, 9,10-ANTH(Br)_2_ (42 mg, 125 µmol) and Na_2_S_2_O_3_ (1.24 g, 5.00 mmol) were dissolved in NMP (10 mL). In a separate Schlenk flask, CF_2_C_6_F_5_I (200 µL, 1.22 mmol) was dissolved in NMP (30 mL). Both solutions were degassed via a freeze-pump-thaw technique (3 × 10 min). The 100 mL Schlenk flask was heated to 160 °C after which the CF_2_C_6_F_5_I solution was added dropwise through a drip funnel. Afterwards, the reaction mixture was kept at 160 °C for 4 h. After letting the reaction mixture cool to room temperature, the reaction mixture was diluted with DCM (50 mL) and washed with H_2_O (6 × 10 mL), brine (1 × 10 mL), and dried over MgSO_4_. The solvents were removed in vacuo. Yield (NMR): 9-Br-10-Bn_F_-ANTH (39%), 9,10-(Bn_F_)_2_-ANTH (<1%).

#### Method 2 (Grignard, solution-phase reactions)

Dry Mg turnings (excess) were added to an oven-dried 100 mL Schlenk flask and mechanically activated via stirring and heating. After 1 h, dry THF (20 mL) and 9,10-ANTH(Br)_2_ (84 mg, 0.25 mmol) were added. Several activation methods were tried (ultra sonic bath, careful heating, addition of I_2_ crystals, addition of a drop of 1,2-dibromoethane). Only the addition of 1,2-dibromoethane enabled the generation of the Grignard reagent which was observed by bubbles and an instant increase of the reaction mixture’s temperature. CF_2_C_6_F_5_I (97.5 µL, 625 µmol) dissolved in dry THF (20 mL) was added with a drip funnel. The reaction mixture was stirred at room temperature for 3 h. It was then washed with HCl (2 × 20 mL), H_2_O (3 × 20 mL), and brine (2 × 20 mL), and extracted with DCM (100 mL). All solvents were removed under vacuum. No conversion could be observed by NMR.

#### Method 3 (photochemical, solution-phase reactions)

CF_2_C_6_F_5_I (63 µL, 0.38 mmol) was syringed into a Schlenk flask containing 9,10-ANTH(Br)_2_ (13.4 mg, 39.8 µmol) and 1,8-diazabicyclo(5.4.0)undec-7-ene (60 µL, 40.1 µmol) dissolved in acetone (technical grade), and degassed three times by a freeze-pump-thaw technique (3 × 10 min). The Schlenk flask was then placed in a metal container equipped with a blue LED strip (1.2 W, Tenmiro) at room temperature for 20 h. Afterwards, the reaction mixture was washed with a 10% solution of Na_2_S_2_O_3_ (2 × 10 mL), H_2_O (3 × 10 mL), and brine (1 × 10 mL) before being dried over MgSO_4_. All solvents were removed in vacuo. Yield (NMR): 9-Br-10-Bn_F_-ANTH (22%), 9,10-(Bn_F_)_2_-ANTH (45%).

#### Product characterization and NMR spectra

9-ANTH(Bn_F_): white/yellow solid; isolated yield 14% based on ANTH; mp 177–179 °C; ^19^F NMR (CDCl_3_, δ/ppm): −73.61 (t, *J* = 17.7 Hz, 2F), −140.94 (m, 2F), −152.56 (t, *J* = 20.4 Hz, 1F), −163.31 (m, 2F); ^1^H NMR (δ/ppm): 8.63 (s, 1H), 8.34 (d, *J* = 7.83 Hz, 2H), 8.06 (d, *J* = 7.72 Hz, 2H), 7.49 (m, 4H); EIMS (*m*/*z*): [M − H]^+^ calcd for 393.051; found, 393.120 (exp),

9,10-ANTH(Bn_F_)_2_: white/yellow solid; isolated yield 17% based on ANTH; mp 211–214 °C; ^19^F NMR (CDCl_3_, δ/ppm): −71.73 (t, *J* = 13.63 Hz, 2F), −140.67 (m, 2F), −151.7 (t, *J* = 21.8 Hz, 1F), −162.68 (m, 2F); ^1^H NMR (δ/ppm): 8.23 (m, 4H), 7.45 (m, 4H); EIMS (*m*/*z*): [M]^+^ calcd for 610.0402; found, 610.100.

### Absorption and emission spectroscopy

Absorption spectra were collected under aerobic conditions in cyclohexane. Measurements were repeated three times and averaged. Emission spectra were collected between 300 and 800 nm with an emission step of 2.000 nm at 1.0 seconds per step, emission bandwidth of 2.000 nm. The samples were degassed using a freeze-pump-thaw technique. A blank of pure cyclohexane was also measured and used to correct the fluorescence spectra. The absorbance of the standard and sample were matched at the excitation wavelength and the absorbance at and above the excitation wavelength was kept below 0.1. The temperature was held constant at 25.0 ± 0.2 °C. The excitation wavelength used was 333 nm. The concentrations used for ANTH, 9,10-ANTH(Bn_F_)_2_, and quinine sulfate were 1.89, 1.14, and 1.94 (10^−5^ M), respectively. Relative quantum yields were calculated using the following equation referenced to quinine sulfate (Φ_f_ = 0.55 in 0.1 M H_2_SO_4_):


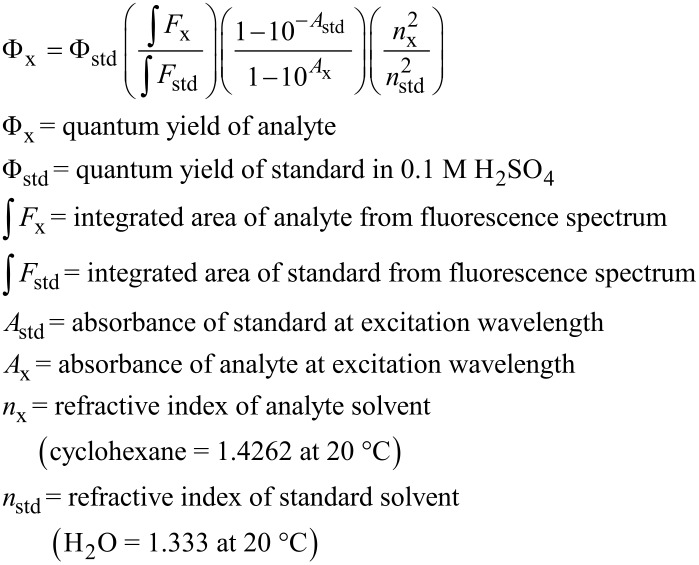


### X-ray crystallography

The diffraction-quality single crystals of 9-10-bis(perfluorobenzyl)anthracene were mounted in a paratone oil on glass fiber rods glued to a small copper wire. X-ray diffraction data were collected at ChemMatCARS (CARS = consortium for advanced radiation sources) sector 15-B at the Advanced Photon Source (Argonne National Laboratory). The data were collected at 100(2) K using a diamond (111) crystal monochromator, a wavelength of 0.41328 Å, and a Bruker CCD detector. The structure was solved using direct methods and refined (on *F*^2^, using all data) by a full-matrix, weighted least squares process. Standard Bruker control and integration software (APEX II) was employed [38], and Bruker SHELXTL software [39] was used for structure solution, refinement, and graphics.

Crystal data for 9,10-ANTH(Bn_F_)_2_: C_28_H_8_F_14_, *M* = 610.34, orthorhombic, *a* = 11.5338(4) Å, *b* = 24.6701(9) Å, *c* = 8.0275(3) Å, α = 90°, β = 90°, γ = 90°, *V* = 2284.15 Å^3^, *T* = 100(2) K, space group *Aba*2, *Z* = 4, synchrotron radiation at ChemMatCARS Sector 15-B at the Advanced Photon Source at Argonne National Laboratory (diamond (111) crystal monochromator μ(diamond (111)) = 0.073 mm^−1^; λ = 0.41328 Å). 15960 reflections measured, 2395 independent reflections (*R*_int_ = 0.0356). The final *R*_1_ values were 0.0241 (*I* > 2σ(*I*)) and 0.0245 (all data). The final *wR*(*F*^2^) values were 0.0613 (*I* > 2σ(*I*)) and 0.0616 (all data). The goodness of fit on *F*^2^ was 1.054. The CCDC number is 1407453.

## Supporting Information

File 1 Experimental details, spectroscopic data, and crystallographic data.

## Data Availability

Data generated and analyzed during this study is available from the corresponding author upon reasonable request.
