# Supplementary material for: Deep-blue emitting 9,10-bis(perfluorobenzyl)anthracene
Source: Beilstein J Org Chem. 2025 Mar 7;21:515–25. doi: 10.3762/bjoc.21.39 (PMC11897650; doi:10.3762/bjoc.21.39)
Supplement: File 1 — Experimental details, spectroscopic data, and crystallographic data. [file Beilstein_J_Org_Chem-21-515-s001.pdf]

BEILSTEIN JOURNAL OF ORGANIC CHEMISTRY

Supporting Information

for

Deep-blue emitting 9,10-bis(perfluorobenzyl)anthracene

Long K. San, Sebastian Balser, Brian J. Reeves, Tyler T. Clikeman, Yu-Sheng Chen, Steven H. Strauss and Olga V. Boltalina

Beilstein J. Org. Chem. **2025,** *21,* 515–525. doi:10.3762/bjoc.21.39

Experimental details, spectroscopic data, and crystallographic data

License and Terms: This is a supporting information file under the terms of the Creative Commons Attribution License (https://creativecommons.org/licenses/by/4.0). Please note that the reuse, redistribution and reproduction in particular requires that the author(s) and source are credited and that individual graphics may be subject to special legal provisions.
The license is subject to the *Beilstein Journal of Organic Chemistry* terms and conditions: (https://www.beilstein-journals.org/bjoc/terms)

Table of contents

Figure S1: Emission spectrum of the blue LED strip S2
Figure S2: ^{1}H NMR spectra of the reaction mixture starting from 9,10-ANTH(Br)$_2$ S3
Figure S3: HPLC separation scheme for 9-ANTH(Bn$_F$) and 9,10-ANTH(Bn$_F$)$_2$ S4
Figure S4: ^{19}F NMR spectrum of 9-ANTH(Bn$_F$), ^{1}H NMR spectra of 9-ANTH(Bn$_F$) and 9,10-ANTH(Bn$_F$)$_2$ S5
Figure S5: Solid-state packing of 9,10-ANTH(Bn$_F$)$_2$ S8
Figure S6: Absorption and emission spectra of ANTH and 9,10-ANTH(Bn$_F$)$_2$ S9
Figure S7: Absorbance decay over time for ANTH and 9,10-ANTH(Bn$_F$)$_2$ S9
Figure S8: ^{1}H NMR spectra of ANTH and 9,10-ANTH(Bn$_F$)$_2$ post photostability S10
Figure S9: ^{19}F NMR spectrum of 9,10-ANTH(Bn$_F$)$_2$ post photostability S11
Figure S10: HPLC chromatogram of ANTH and 9,10-ANTH(Bn$_F$)$_2$ post photostability S12
Figure S11: ^{19}F NMR spectra of 9,10-ANTH(Bn$_F$)$_2$ anaerobic and aerobic S13
Figure S12: The fluorescence of 9,10-ANTH(Bn$_F$)$_2$ in CH_2Cl_2 S14
Figure S13: DART-MS analysis of photo irradiated 9,10-ANTH(Bn$_F$)$_2$ S14
Table S1: The % remaining during irradiation experiment S15

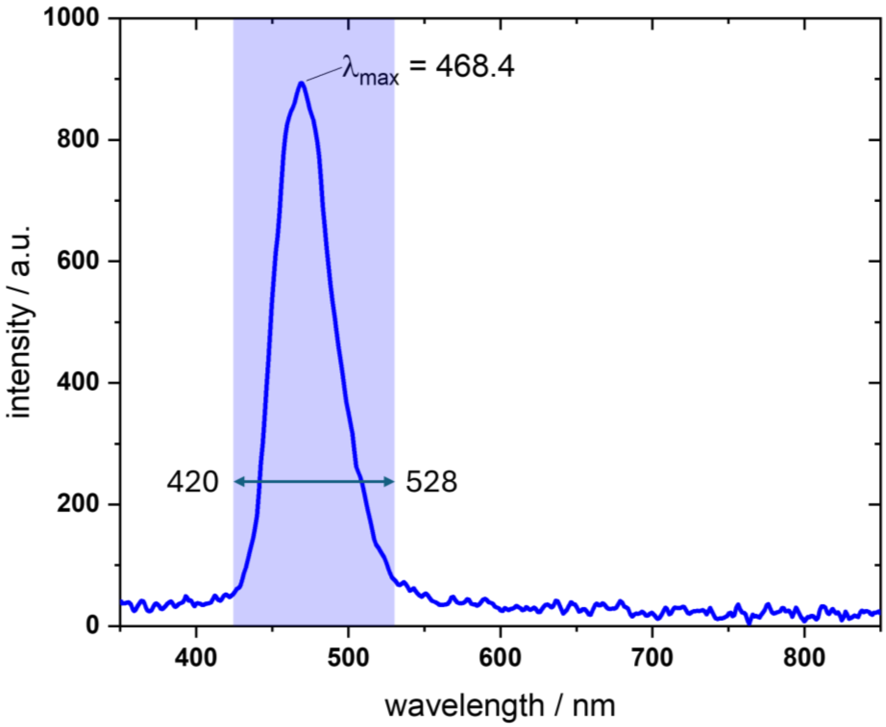

Figure S1: Emission spectrum of the blue LED strip (1.2 W, *Tenmiro*), used for the photochemical conversion of 9,10-ANTH(Br)$_2$ to 9,10-ANTH(Bn$_F$)$_2$.

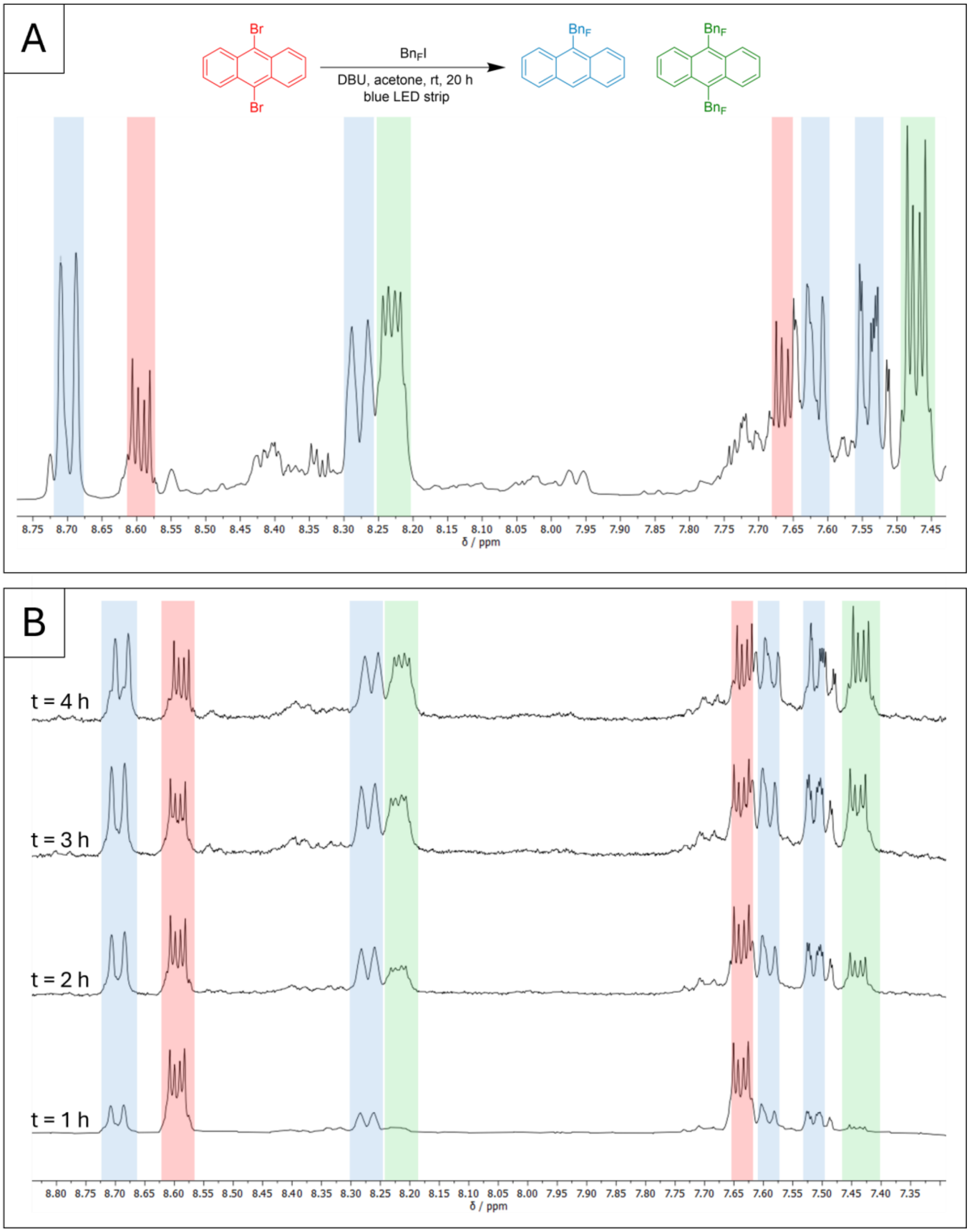

Figure S2: A: ^{1}H NMR spectra of the crude reaction mixture at the end of the reaction (20 h) showing the signals of 9,10-ANTH$(Br)_2$ (red), 9-ANTH(Bn_F) (blue) and 9,10-ANTH$(Bn_F)_2$ (green) in $CDCl_3$. **B**: ^{1}H NMR spectra of the reaction mixture after 1, 2, 3, and 4 h (procedure: 1 mL from the reaction was taken out of the flask, the solvent was evaporated, and the residue was redissolved in $CDCl_3$).

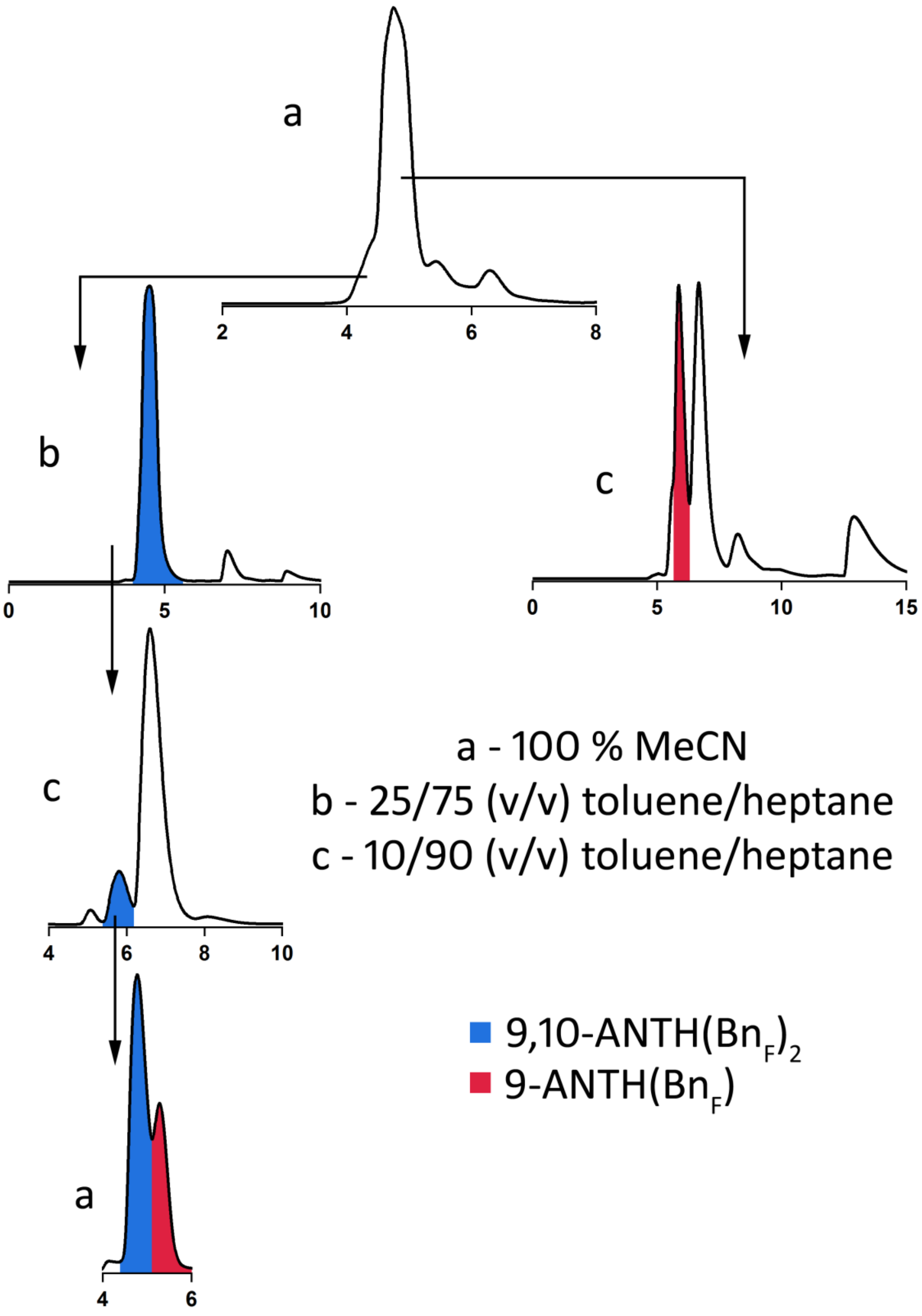

Figure S3: HPLC chromatograms for the separation of 9,10-ANTH(Bn_F)$_2$ and 9-ANTH(Bn_F). The conditions are listed next to the chromatograms. All traces collected used a COSMOSIL Buckyprep semi-preparative stationary phase at a flow rate of 5 mL min^{-1} and observed at 300 nm detection wavelength. The peaks in color are those collected and further separated in a subsequent separation stage. The blue color corresponds to 9,10-ANTH(Bn_F)$_2$ and the magenta color corresponds to 9-ANTH(Bn_F). The *x*-axis for all chromatograms is the t_R in minutes. Chromatogram a is of the crude product mixture.

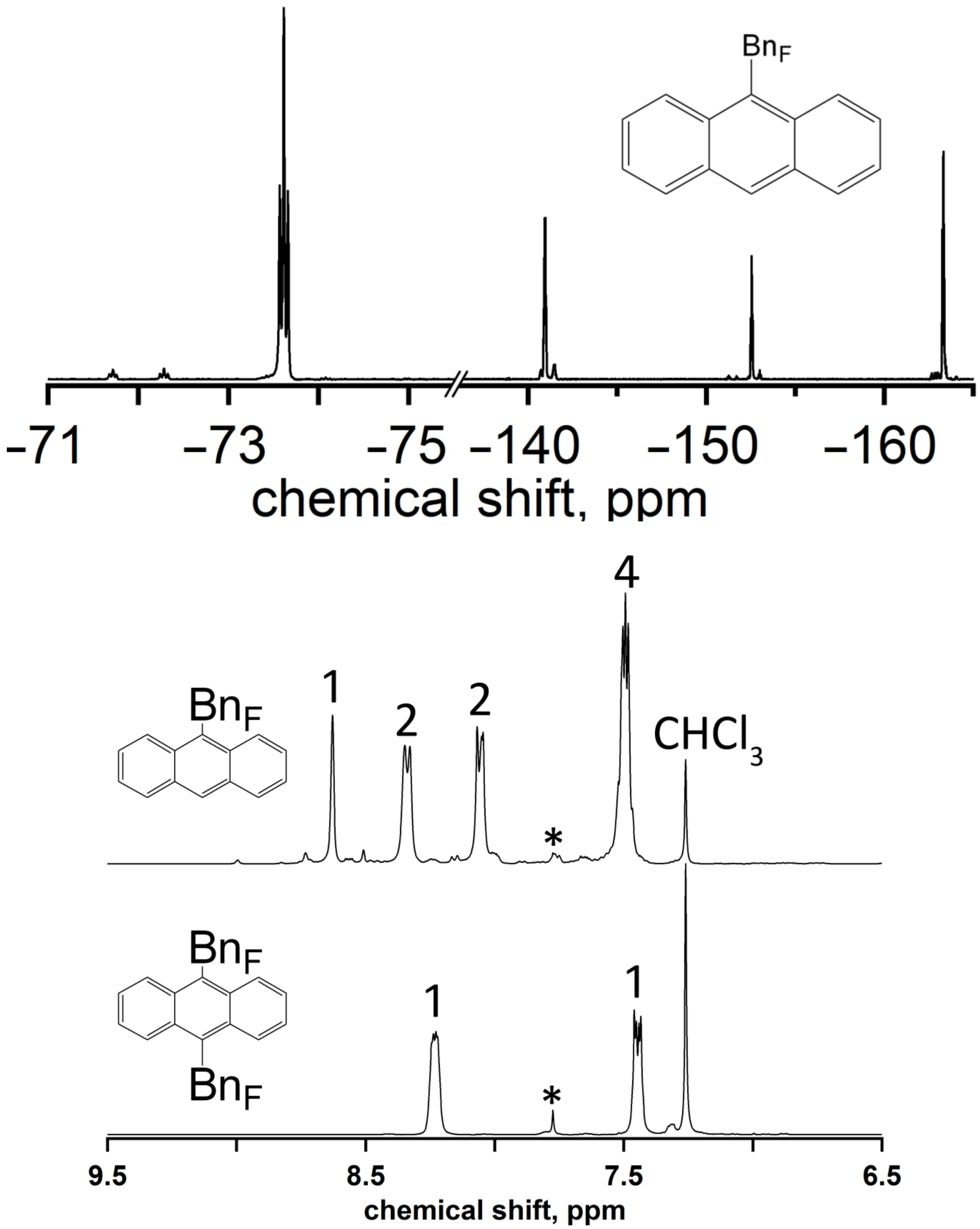

Figure S4: top: ^{19}F NMR spectrum (379 MHz) of 9-ANTH(Bn$_F$) in $CDCl_3$ with molecular structure drawing. **Bottom:** ^{1}H NMR spectra of 9-ANTH(Bn$_F$) and 9,10-ANTH(Bn$_F$)$_2$ in $CDCl_3$ (residual resonance δ 7.26 ppm). The internal standard 1,4-bis(trifluoromethyl)benzene is marked by an asterisk at δ 7.77 ppm).

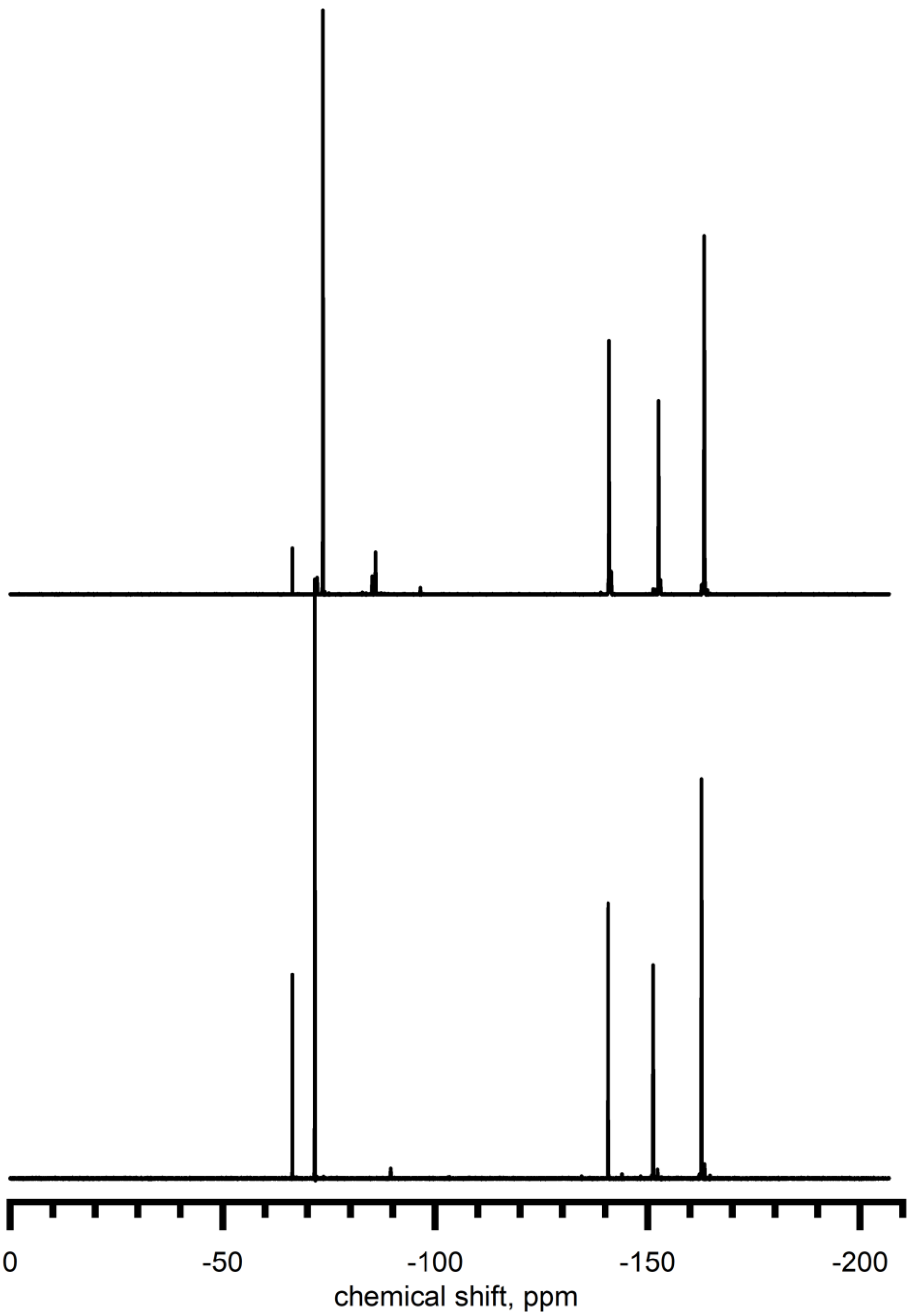
0
-50
-100
-150
-200
chemical shift, ppm

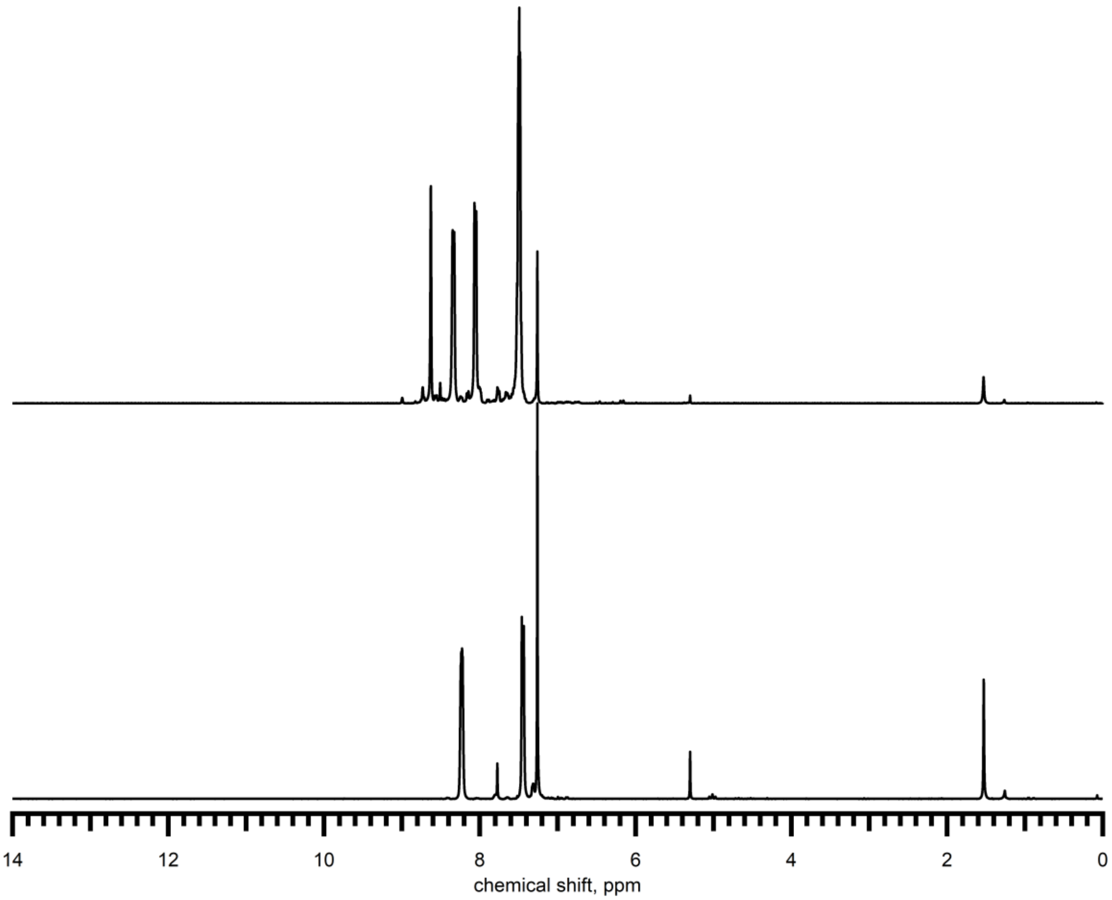

Figure S4a expanded full spectra: top panel: ^{19}F NMR spectra (379 MHz) of 9-ANTH(Bn$_F$) (top) and 9,10-ANTH(Bn$_F$)$_2$ (bottom) in $CDCl_3$. Bottom panel: ^{1}H NMR spectra of 9-ANTH(Bn$_F$) and 9,10-ANTH(Bn$_F$)$_2$ in $CDCl_3$ (residual resonance δ 7.26 ppm).

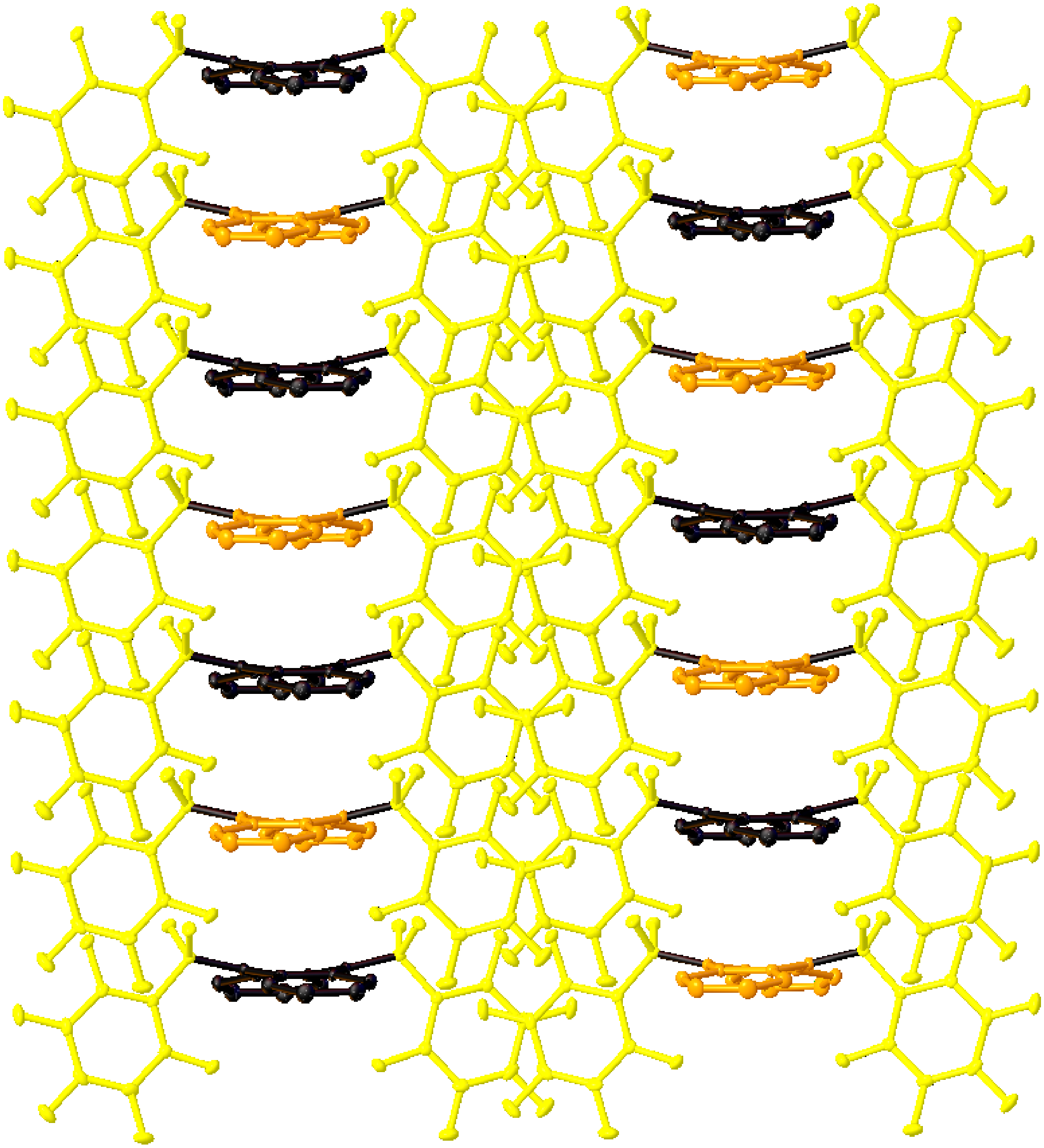

Figure S5: Solid-state packing of 9,10-ANTH(Bn_F)$_2$ viewed along the major axis of ANTH core. Two different stacked columns are colored in orange and black, and the Bn_F groups are colored in yellow. The Bn_F groups are shown to insult the ANTH core. Hydrogen atoms are omitted for clarity.

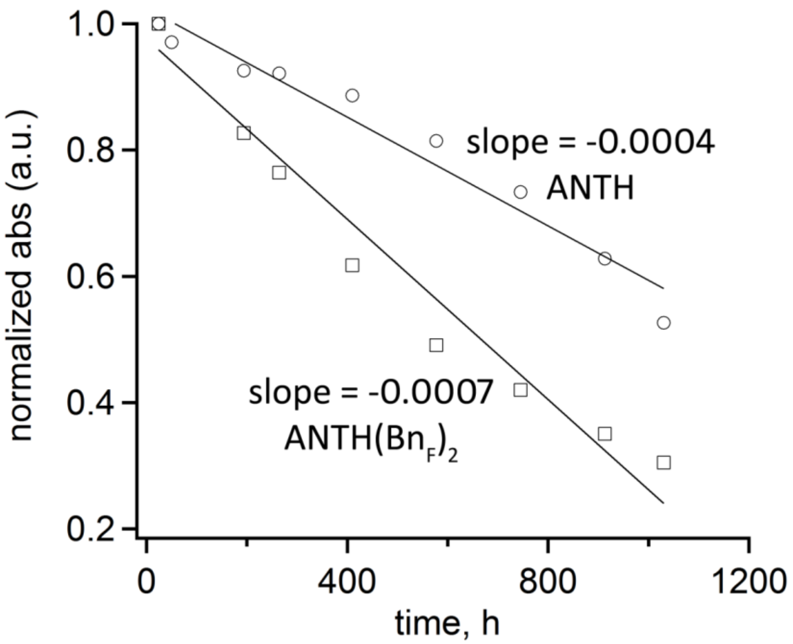

Figure S6: Normalized absorbance decay over time for ANTH and 9,10-ANTH(Bn_F)$_2$ under aerobic conditions and irradiated with 34 W ambient fume hood light bulb. Samples were dissolved in CH_2Cl_2 and placed into a quartz sample holder.

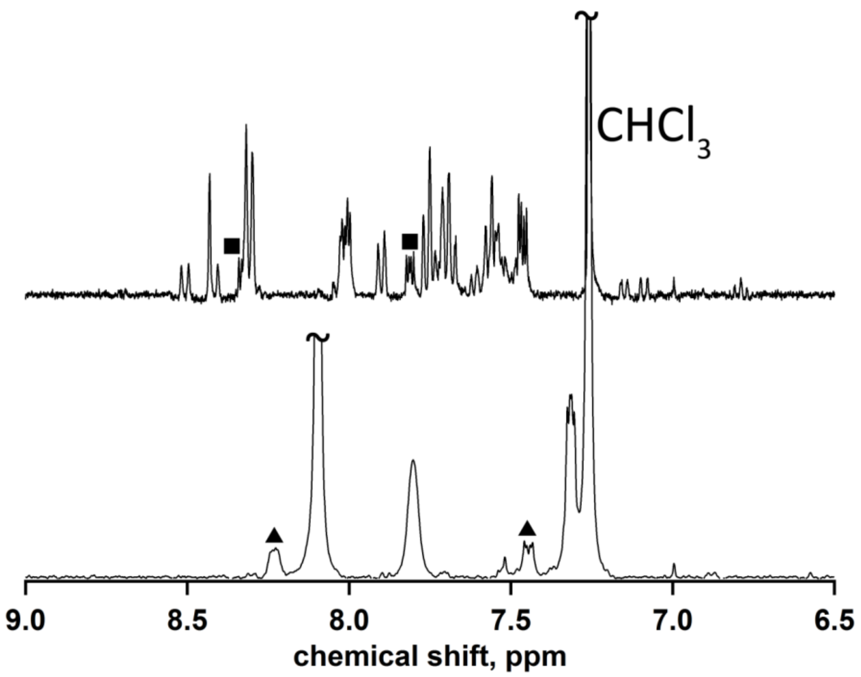

Figure S7: ^{1}H NMR spectra of ANTH (top) and 9,10-ANTH(Bn_F)$_2$ (bottom) post aerobic photostability in $CDCl_3$ (residual resonance δ 7.26 ppm). The black squares represent resonances corresponding to anthraquinone and the black triangles represent resonances corresponding to 9,10-ANTH(Bn_F)$_2$. Unidentified solvent impurity at δ 8.1 ppm.

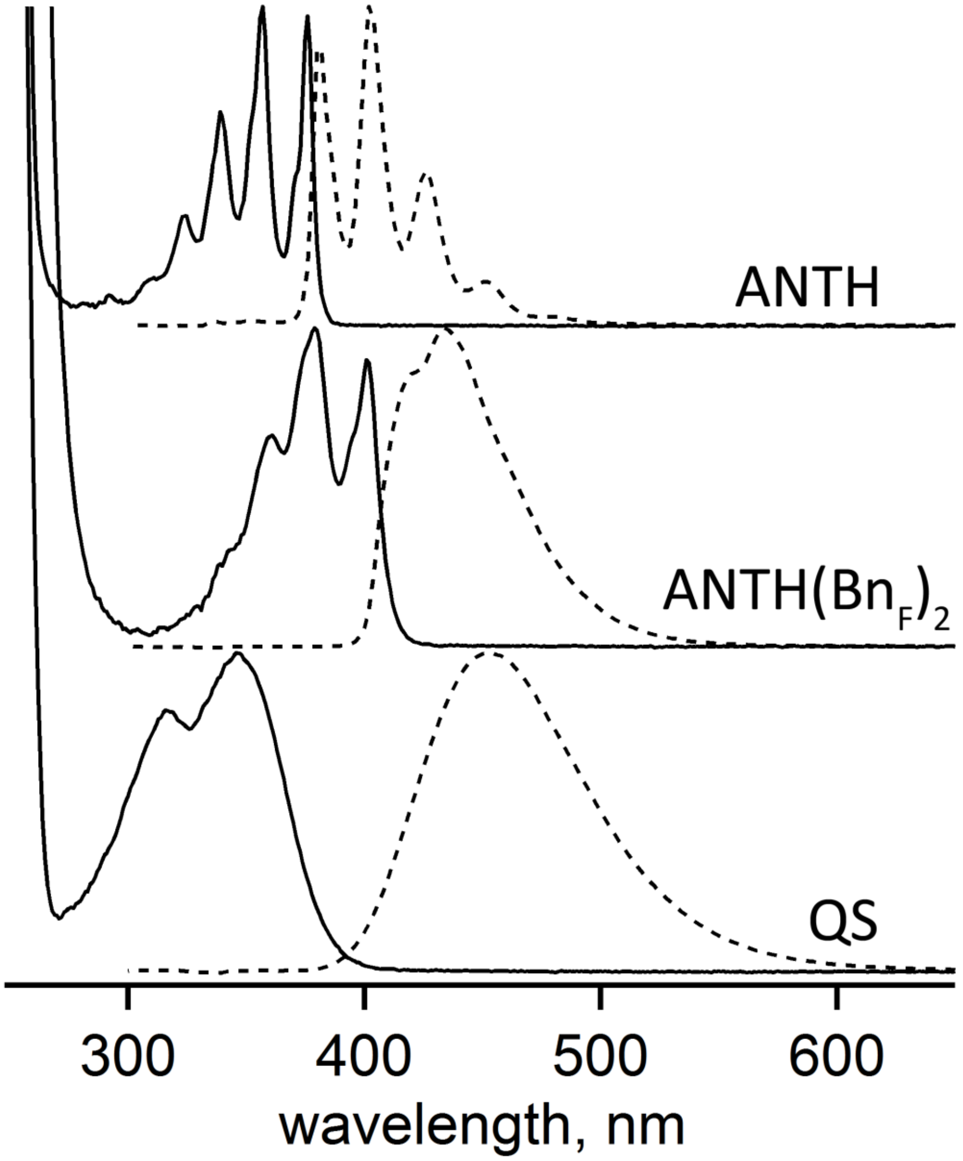

Figure S8: Absorption and emission spectra of ANTH and 9,10-ANTH(Bn_F)$_2$ in cyclohexane, and quinine sulfate (QS) in 0.1 M aqueous H_2SO_4. Samples were under aerobic (absorption, solid line) and anaerobic (emission, dashed line) conditions.

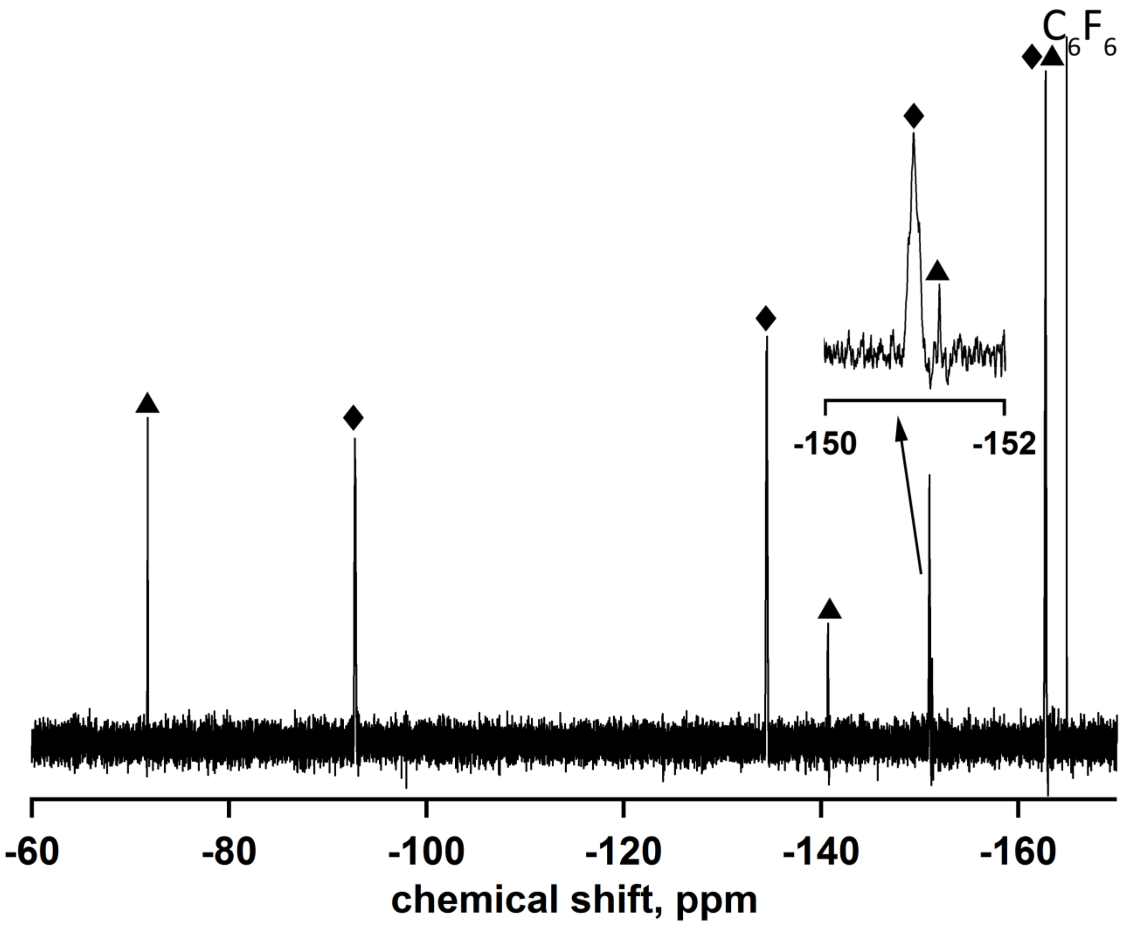

Figure S9: ^{19}F NMR spectrum of 9,10-ANTH(Bn_F)$_2$ post aerobic photostability in $CDCl_3$. The internal standard C_6F_6 (δ −164.9 ppm) is shown. The black triangles represent resonances corresponding to 9,10-ANTH(Bn_F)$_2$ and the black diamonds represent resonances corresponding to the new symmetric compound.

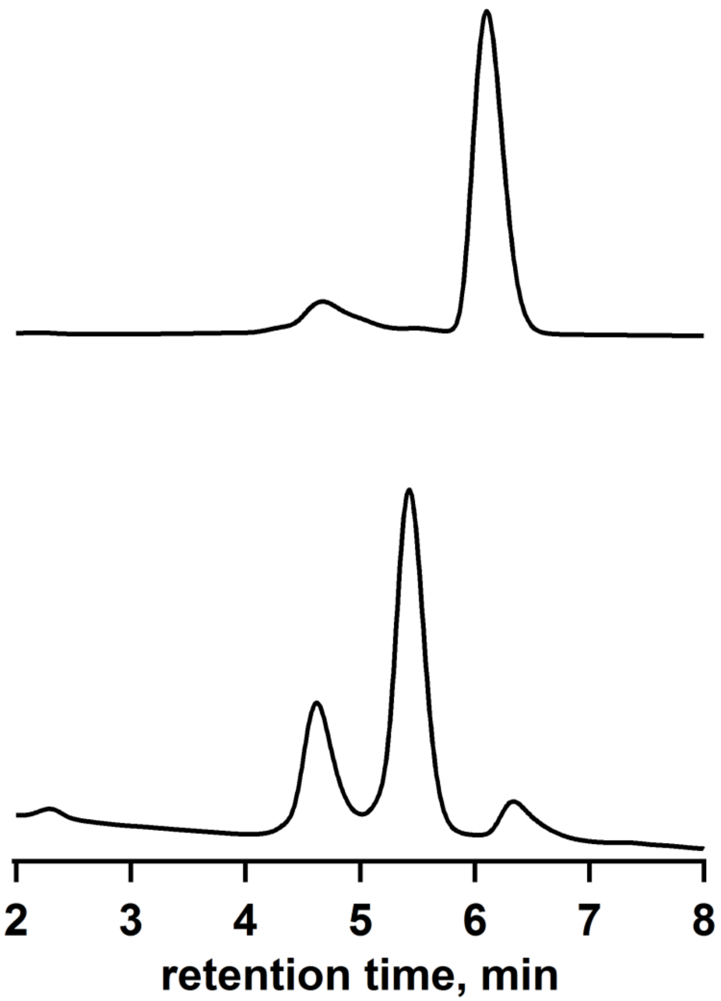

Figure S10: HPLC chromatograms of ANTH (bottom) and 9,10-ANTH(Bn_F)$_2$ (top) post aerobic photostability experiments in 100% MeCN at a flow rate of 5 mL min^{-1}. The compounds ANTH and 9,10-ANTH(Bn_F)$_2$ have retention times of 5.5 minutes and 4.6 minutes, respectively. Note: ANTH co-elutes with anthraquinone (major peak in bottom chromatogram).

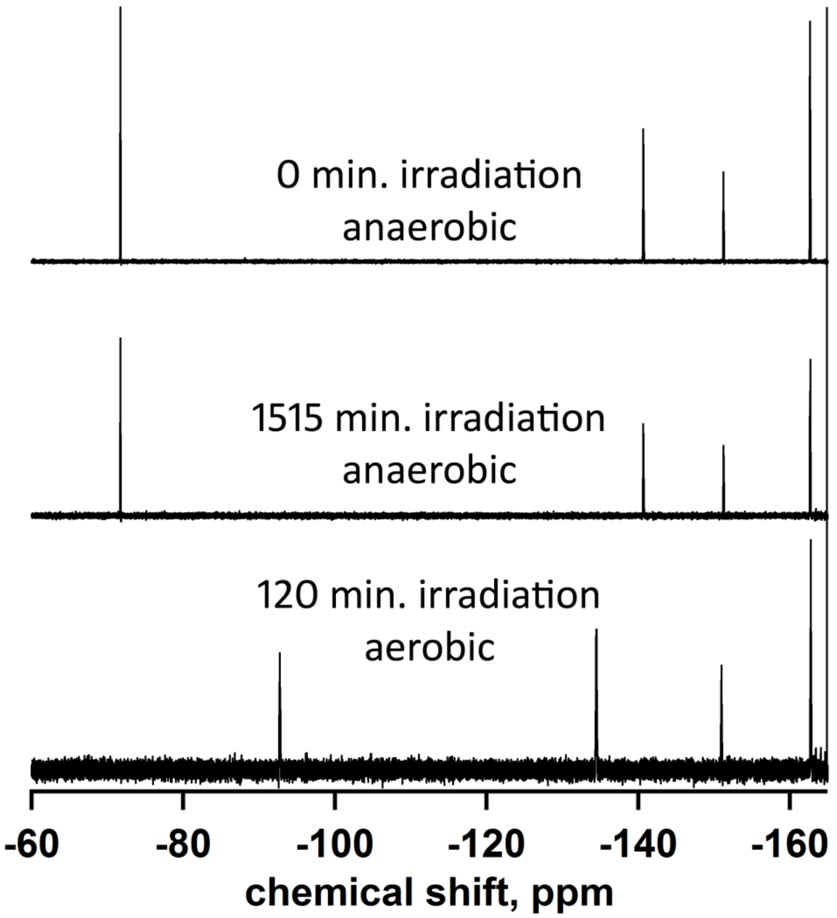

Figure S11: ^{19}F NMR spectra of 9,10-ANTH(Bn_F)$_2$ irradiated for 0 min. (top), 1515 min. (middle), and after exposure to air with additional 120 min irradiation (bottom). Internal standard C_6F_6 shown at δ −164.9 ppm.

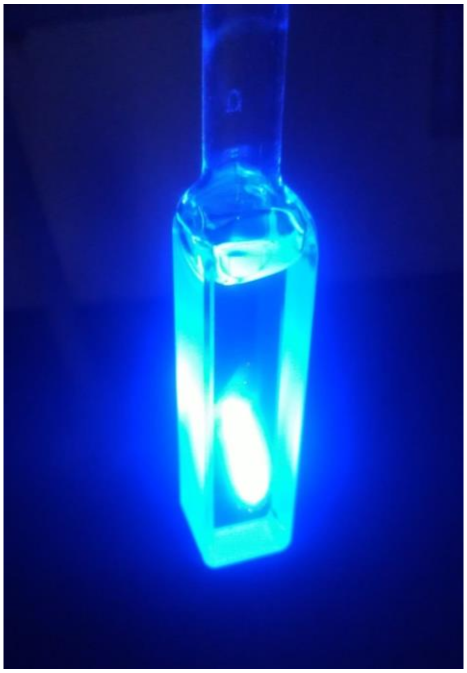

Figure S12: The fluorescence of 9,10-ANTH(Bn_F)$_2$ in CH_2Cl_2 under a 405 nm illumination.

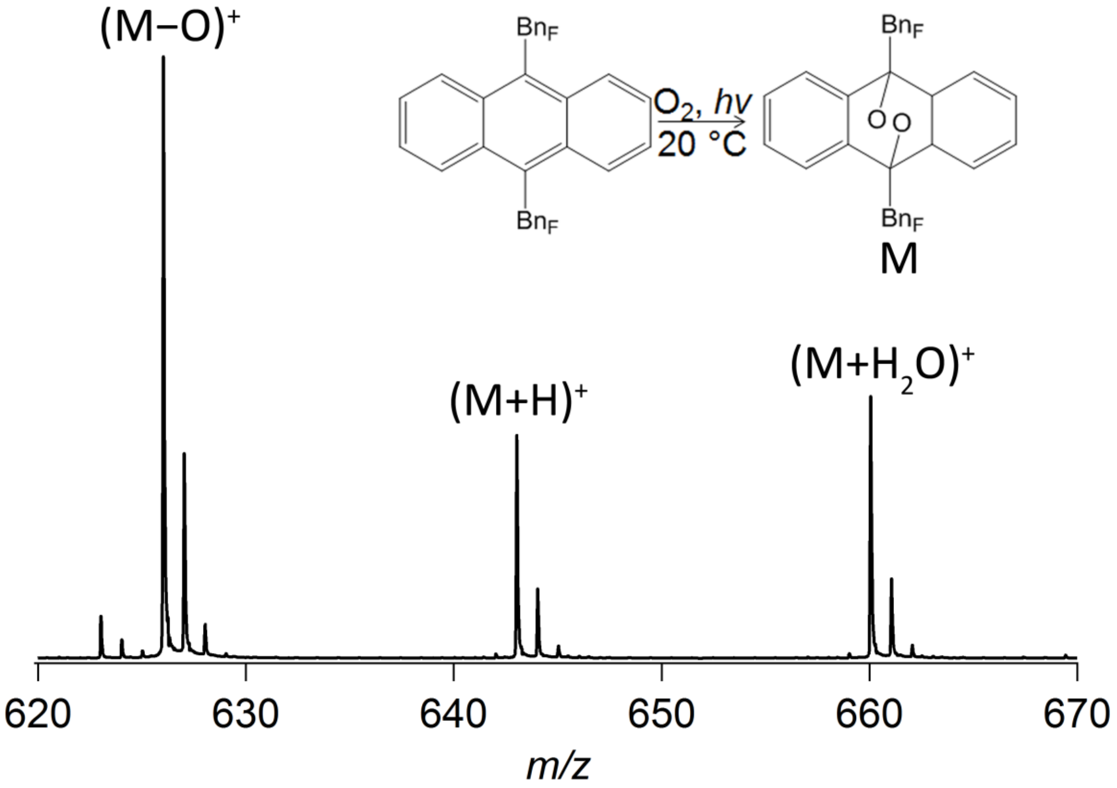

Figure S13: DART-MS analysis of the photo irradiated 9,10-ANTH(Bn_F)$_2$ sample.

time (min)	% remaining, ANTH	% remaining, ANTH(Bn_F)$_2$
30	100.0	100.0
60	85.0	94.6
90	79.4	88.9
180	58.8	85.9
240	54.0	79.3
300	51.2	73.2
540	22.3	50.3
720	20.8	41.5
960	16.3	31.5
1275	13.1	25.5

Table S1: Time of irradiation and % of ANTH and 9,10-ANTH(Bn_F)$_2$ remaining based on the $\delta(^1H)$ 7.48 and $\delta(^1H)$ 7.46, respectively.
